# A phase 1, first‐in‐human, dose‐escalation study of JNJ‐74856665 (dihydroorotate dehydrogenase inhibitor) alone or in combination in patients with AML or MDS

**DOI:** 10.1111/bjh.20224

**Published:** 2025-07-01

**Authors:** Emma Searle, Thomas Cluzeau, Jenny O'Nions, Christian Recher, Emmanuel Gyan, Daniel Morillo, Jordi Esteve, Austin Kulasekararaj, Emmanuel Raffoux, Harriet S. Walter, Arnaud Pigneux, David Valcárcel, Nikki Daskalakis, Jacqueline Bussolari, E. Christine Pietsch, Scott Kuduk, Tammy Bush, Xiaochun Zhang, Ulrike Philippar, Adetokunbo Oluwasanjo, Kathryn Bradford, Jonathan Miller, Charlotte Van Bogaert, Martin Curtis, Shih‐Yu Chang, Christina Guttke, Stan Gaj, Rachel Pearson, Joseph Murphy, Wim van Dijck, Ana Alfonso Piérola

**Affiliations:** ^1^ The Christie NHS Foundation Trust Manchester UK; ^2^ University of Manchester Manchester UK; ^3^ CHU de Nice, Hôpital Archet 1 Nice France; ^4^ University College London Hospitals NHS Foundation Trust London UK; ^5^ Centre Hospitalier Universitaire de Toulouse Institut Universitaire du Cancer de Toulouse Oncopole Toulouse France; ^6^ Hématologie et Thérapie Cellulaire, CIC INSERM U1415 Centre Hospitalier Universitaire de Tours, Université de Tours Tours France; ^7^ Fundación Jiménez Díaz University Hospital START Madrid‐FJD Madrid Spain; ^8^ Hospital Clínic de Barcelona Barcelona Spain; ^9^ King's College Hospital London UK; ^10^ Hôpital Saint‐Louis (AP‐HP) Paris France; ^11^ University of Leicester Leicester UK; ^12^ Centre Hospitalier Universitaire de Bordeaux Hôpital Haut Lévêque Bordeaux France; ^13^ Vall d'Hebron Barcelona Spain; ^14^ Johnson & Johnson Spring House Pennsylvania USA; ^15^ Johnson & Johnson, Research & Development Beerse Belgium; ^16^ Johnson & Johnson Research Triangle Park North Carolina USA; ^17^ Johnson & Johnson High Wycombe UK; ^18^ Cancer Center Clinica Universidad de Navarra Pamplona Spain; ^19^ Present address: PAREXEL International Billerica Massachusetts USA; ^20^ Present address: Taiho Oncology, Inc. Princeton New Jersey USA

**Keywords:** acute myeloid leukaemia, dihydroorotate dehydrogenase, JNJ‐74856665, myelodysplastic syndromes, phase 1

To the Editor,

JNJ‐74856665 is an orally bioavailable, potent and selective dihydroorotate dehydrogenase inhibitor (DHODHi) designed to induce myeloid differentiation and eliminate undifferentiated leukaemia blasts and stem cells.^1^, ^2^ JNJ‐74856665 binding to DHODH promotes myeloblast differentiation, cell cycle arrest and apoptosis. Herein, we detail preclinical studies and a first‐in‐human, open‐label, dose‐escalation, dose‐expansion, phase 1 study of JNJ‐74856665, administered alone or in combination with azacitidine (AZA) or venetoclax (VEN) (Figure S1).

Primary clinical trial objectives were to determine maximum tolerated dose (MTD) and recommended phase 2 dose(s) (RP2D[s]) and to evaluate safety and tolerability. Eligible patients (i.e. ≥18 years old with newly diagnosed acute myeloid leukaemia [AML]/myelodysplastic syndromes (MDS),^3^ relapsed/refractory [R/R] AML,^4^ R/R MDS^4^, ^5^ or R/R chronic myelomonocytic leukaemia group 2^4^ [CMML‐2; Table S1]) were enrolled into one of two JNJ‐74856665 monotherapy arms (mono‐R/R or mono‐LR) or one of two combination arms (JNJ+AZA or JNJ+VEN). Detailed methods and results for both the preclinical and clinical studies are in the Supporting Information.

Clinical trial enrolment included 153 patients (218 screened): 84 in mono‐R/R, 35 in JNJ+AZA, 29 in JNJ+VEN and 5 in mono‐LR (Table 1; Tables S3–S5; Figure S6). At data cut‐off, 46 patients remained on study (26 on treatment). Median (range) duration of JNJ‐74856665 treatment was 52 (3–442) days, and number of treatment cycles was 3 (range, 1–22) for mono‐R/R, 7 (2–15) for mono‐LR, 2 (1–13) for JNJ+AZA and 2 (1–7) for JNJ+VEN.

**TABLE 1:** Patient demographic and clinical characteristics (all‐treated/safety population).

	Mono‐R/R	JNJ+AZA	JNJ+VEN	Mono‐LR	Total
*n*	84	35	29	5	153
Age, years					
Mean (SD)	71.6 (9.79)	69.4 (7.39)	70.1 (11.09)	71.4 (6.31)	70.8 (9.45)
Median (range)	73.5 (40–88)	72 (54–80)	74 (29–82)	72 (62–78)	73 (29–88)
<65, *n* (%)	17 (20.2)	10 (28.6)	8 (27.6)	1 (20.0)	36 (23.5)
≥65, *n* (%)	67 (79.8)	25 (71.4)	21 (72.4)	4 (80.0)	117 (76.5)
Sex, *n* (%)					
Female	29 (34.5)	15 (42.9)	7 (24.1)	1 (20.0)	52 (34.0)
Male	55 (65.5)	20 (57.1)	22 (75.9)	4 (80.0)	101 (66.0)
Race, *n* (%)					
Asian	9 (10.7)	9 (25.7)	1 (3.4)	1 (20.0)	20 (13.1)
White	61 (72.6)	24 (68.6)	22 (75.9)	4 (80.0)	111 (72.5)
Unknown	14 (16.7)	2 (5.7)	6 (20.7)	0 (0.0)	22 (14.4)
Ethnicity, *n* (%)					
Hispanic or Latino	30 (35.7)	3 (8.6)	3 (10.3)	0 (0.0)	36 (23.5)
Not Hispanic or Latino	42 (50.0)	29 (82.9)	18 (62.1)	4 (80.0)	93 (60.8)
Not reported	11 (13.1)	1 (2.9)	6 (20.7)	0 (0.0)	18 (11.8)
Diagnosis, *n* (%)					
AML	66 (78.6)	20 (57.1)	24 (82.8)	0 (0.0)	110 (71.9)
De novo	30 (35.7)	12 (34.3)	12 (41.4)	0 (0.0)	54 (35.3)
Secondary	34 (40.5)	7 (20.0)	11 (37.9)	0 (0.0)	52 (34.0)
Therapy related	2 (2.4)	1 (2.9)	0 (0.0)	0 (0.0)	3 (2.0)
Prior MDS	28 (33.3)	6 (17.1)	8 (27.6)	0 (0.0)	42 (27.5)
Prior MPN	4 (4.8)	0 (0.0)	3 (10.3)	0 (0.0)	7 (4.6)
Unknown	2 (2.4)	1 (2.9)	1 (3.4)	0 (0.0)	4 (2.6)
Mutated *TP53* ^a^	10 (16.9)	2 (10.0)	2 (10.5)	0 (0.0)	14 (14.3)
With myelodysplasia‐related changes	34 (40.5)	9 (25.7)	12 (41.4)	0 (0.0)	55 (35.9)
Primary refractory disease	33 (39.3)	11 (31.4)	11 (37.9)	2 (40.0)	57 (37.3)
MDS	18 (21.4)	14 (40.0)	5 (17.2)	5 (100.0)	42 (27.5)
High risk	15 (17.9)	10 (28.6)	5 (17.2)	0 (0.0)	30 (19.6)
Very high risk	3 (3.6)	3 (8.6)	0 (0.0)	0 (0.0)	6 (3.9)
CMML‐2	0 (0.0)	1 (2.9)	0 (0.0)	0 (0.0)	1 (0.7)
Number of lines of prior therapy, *n* (%)					
0	0 (0.0)	9 (25.7)	0 (0.0)	2 (40.0)	11 (7.2)
1	41 (48.8)	9 (25.7)	10 (34.5)	1 (20.0)	61 (39.9)
2	18 (21.4)	9 (25.7)	9 (31.0)	0 (0.0)	36 (23.5)
3	16 (19.0)	4 (11.4)	8 (27.6)	0 (0.0)	28 (18.3)
4+	9 (10.8)	4 (11.4)	2 (6.9)	2 (40.0)	17 (11.1)
Mean (SD)	2.01 (1.3)	2.19 (1.2)	2.07 (1.0)	3.67 (2.5)	2.09 (1.3)
Median (range)	2 (1.0–7.0)	2 (1.0–5.0)	2 (1.0–4.0)	4 (1.0–6.0)	2 (1.0–7.0)
Bone marrow blasts at baseline (fraction of 1), %					
*n*	83	34	28	4	149
Mean (SD)	0.36 (0.253)	0.29 (0.264)	0.34 (0.257)	0.02 (0.020)	0.33 (0.026)
Median (range)	0.29 (0.1–1.0)	0.17 (0.0–0.9)	0.27 (0.0–0.9)	0.01 (0.0–0.1)	0.25 (0.0–1.0)
Absolute peripheral blasts at baseline, × 10^9^/L					
*n*	34	12	11	2	59
Mean (SD)	1.95 (4.17)	1.19 (2.89)	0.88 (1.25)	0.07 (0.09)	1.53 (3.47)
Median (range)	0.29 (0.0–15.6)	0.15 (0.00–9.81)	0.32 (0.00–3.48)	0.07 (0.00–0.13)	0.20 (0.00–15.60)
Duration of treatment, days^b^					
Mean (SD)	69.6 (72.5)	96.1 (90.2)	64.8 (46.4)	151.4 (97.7)	77.5 (75.25)
Median (range)	50.0 (3.0–442.0)	54.0 (3.0–362.0)	51.0 (5.0–170.0)	147.0 (23.0–295.0)	52.0 (3.0–442.0)

*Note*: Percentages were calculated based on the number of patients in each group as denominator. A patient was considered as treated in a cycle if they received any nonzero dose of JNJ‐74856665 in that cycle. The interval from the last prior line of therapy was defined as the time between the treatment start date and the date of the last prior line of therapy. Mono‐R/R and JNJ+VEN: patients with R/R AML who had exhausted or were ineligible for standard therapeutic options, patients with newly transformed secondary AML who exhausted standard therapeutic options during treatment before transformation, and patients with high risk or very high risk, R/R MDS who exhausted or were ineligible for standard therapeutic options. The mono‐R/R arm also was to include patients with R/R CMML‐2 who exhausted or were ineligible for standard therapeutic options. JNJ+AZA: patients with newly diagnosed or R/R AML who were unsuitable for intensive treatment with a curative intent (including stem cell transplantation) but eligible to receive AZA, patients with high‐risk or very high‐risk MDS‐and patients with CMML‐2. Mono‐LR: patients with very low‐, low‐ or intermediate‐risk MDS and transfusion dependence.

Abbreviations: AML, acute myeloid leukaemia; AZA, azacitidine; CMML‐2, chronic myelomonocytic leukaemia group 2; JNJ, JNJ‐74856665; LR, lower risk; MDS, myelodysplastic syndromes; MPN, myeloproliferative neoplasms; R/R, relapsed or refractory; SD, standard deviation; *TP53*, tumour protein 53; VEN, venetoclax.

^a^ Among patients with AML and available molecular testing data (*n* = 101; 9 patients with AML did not have available molecular testing data).

^b^ Total duration of treatment was defined as (date of last dose of JNJ‐74856665 − date of first dose of JNJ‐74856665) + 1.

Dose escalation was stopped based on limited clinical activity after reviewing available safety, pharmacokinetic/pharmacodynamic and efficacy data. MTD was not reached, and RP2D was not established.

Most (97.4%) patients experienced ≥1 treatment‐emergent adverse event (TEAE), 64.7% of which were considered JNJ‐74856665 related by investigators (Table S6). The most common TEAEs were stomatitis (48.8%; all JNJ‐74856665 related), diarrhoea (32.1%; 17.9% JNJ‐74856665 related), anaemia (31%) and asthenia (25%) in the mono‐R/R arm; mouth ulceration (40%) in the mono‐LR arm; thrombocytopenia and vomiting (42.9% each), diarrhoea (37.1%), nausea (34.3%), anaemia and constipation (31.4% each) and stomatitis (20.0%; 17.1% JNJ‐74856665 related) with JNJ+AZA; and diarrhoea (27.6%), neutropenia (27.6%) and stomatitis (13.8%) with JNJ+VEN. TEAEs in ≥10% of patients are in Table 2. In the mono‐R/R, JNJ+AZA and JNJ+VEN arms, 82.1%, 94.3% and 86.2% of patients, respectively, experienced Grade ≥3 TEAEs (JNJ‐74856665 related in 34.5%, 31.4% and 27.6% of patients) (Table S6). Furthermore, 72.6%, 71.4% and 75.9% of patients, respectively, experienced SAEs (JNJ‐74856665 related in 19.0%, 17.1% and 13.8%) (Table S7).

**TABLE 2:** Treatment‐emergent adverse events with a frequency of ≥10%, overall and related to JNJ‐74856665 (safety population).

	Mono‐R/R	JNJ+AZA	JNJ+VEN	Mono‐LR
*n*	84	35	29	5
Patients with ≥1 TEAE with a frequency of ≥10%, *n* (%)	81 (96.4)	35 (100.0)	28 (96.6)	5 (100.0)
Gastrointestinal disorders	68 (81.0)	30 (85.7)	18 (62.1)	3 (60.0)
Stomatitis	41 (48.8)	7 (20.0)	4 (13.8)	—
Diarrhoea	27 (32.1)	13 (37.1)	8 (27.6)	1 (20.0)
Nausea	12 (14.3)	12 (34.3)	5 (17.2)	—
Vomiting	12 (14.3)	15 (42.9)	—	—
Constipation	—	11 (31.4)	3 (10.3)	—
Abdominal pain	—	4 (11.4)	—	—
Haemorrhoids	—	4 (11.4)	—	—
Mouth ulcerations	—	—	—	2 (40.0)
Aphthous ulcer	—	—	—	1 (20.0)
Odynophagia	—	—	—	1 (20.0)
Blood and lymphatic system disorders	40 (47.6)	21 (60.0)	15 (51.7)	1 (20.0)
Thrombocytopenia	20 (23.8)	15 (42.9)	6 (20.7)	—
Anaemia	26 (31.0)	11 (31.4)	7 (24.1)	—
Neutropenia	10 (11.9)	8 (22.9)	8 (27.6)	1 (20.0)
Febrile neutropenia	—	—	4 (13.8)	—
General disorders and administration site conditions	40 (47.6)	26 (74.3)	15 (51.7)	—
Asthenia	21 (25.0)	7 (20.0)	—	—
Pyrexia	13 (15.5)	6 (17.1)	5 (17.2)	—
General physical health deterioration	9 (10.7)	—	3 (10.3)	—
Injection site reaction	—	8 (22.9)	—	—
Fatigue	—	6 (17.1)	3 (10.3)	—
Oedema peripheral	—	—	3 (10.3)	—
Infections and infestations	51 (60.7)	25 (71.4)	20 (69.0)	1 (20.0)
Bronchopulmonary aspergillosis	—	4 (11.4)	—	—
Pneumonia	12 (14.3)	8 (22.9)	3 (10.3)	—
COVID‐19	—	—	7 (24.1)	—
Gastroenteritis norovirus	—	—	—	1 (20.0)
Klebsiella infection	—	—	—	1 (20.0)
Investigations	13 (15.5)	—	6 (20.7)	1 (20.0)
Lipase increased	—	—	—	—
Alanine aminotransferase increased	—	—	—	1 (20.0)
Respiratory, thoracic and mediastinal disorders	30 (35.7)	13 (37.1)	7 (24.1)	1 (20.0)
Epistaxis	10 (11.9)	—	—	—
Cough	—	4 (11.4)	3 (10.3)	—
Dyspnoea	—	4 (11.4)	—	1 (20.0)
Skin and subcutaneous tissue disorders	25 (29.8)	17 (48.6)	3 (10.3)	1 (20.0)
Hidradenitis	—	—	—	1 (20.0)
Rash maculopapular	—	4 (11.4)	—	—
Metabolism and nutrition disorders	29 (34.5)	9 (25.7)	9 (31.0)	—
Decreased appetite	10 (11.9)	4 (11.4)	—	—
Musculoskeletal and connective tissue disorders	15 (17.9)	7 (20.0)	—	—
Vascular disorders	15 (17.9)	6 (17.1)	4 (13.8)	—
Hypotension	—	5 (14.3)	—	—
Injury, poisoning and procedural complications	11 (13.1)	6 (17.1)	3 (10.3)	—
Nervous system disorders	11 (13.1)	9 (25.7)	8 (27.6)	—
Headache	—	4 (11.4)	4 (13.8)	—
Dizziness	—	—	3 (10.3)	—
Cardiac disorders	10 (11.9)	4 (11.4)	3 (10.3)	—
Eye disorders	—	4 (11.4)	—	—
Renal and urinary disorders	9 (10.7)	—	3 (10.3)	—
Patients with ≥1 TEAE related to JNJ‐74856665 with a frequency of ≥10%, *n* (%)	60 (71.4)	23 (65.7)	13 (44.8)	3 (60.0)
Gastrointestinal disorders	54 (64.3)	19 (54.3)	10 (34.5)	2 (40.0)
Stomatitis	41 (48.8)	6 (17.1)	4 (13.8)	
Diarrhoea	15 (17.9)	8 (22.9)	3 (10.3)	1 (20.0)
Nausea	—	6 (17.1)	—	—
Aphthous ulcer	—	—	—	1 (20.0)
Odynophagia	—	—	—	1 (20.0)
Blood and lymphatic system disorders	13 (15.5)	7 (20.0)	3 (10.3)	—
Thrombocytopenia	11 (13.1)	5 (14.3)	3 (10.3)	—
Skin and subcutaneous tissue disorders	10 (11.9)	—	—	—
General disorders and administration site conditions	—	6 (17.1)	—	—
Fatigue	—	4 (11.4)	—	—
Infections and infestations	—	5 (14.3)	—	—

*Note*: AE relationship was specifically concerned with JNJ‐74856665. An AE was assessed by the investigator as related to study agent. Patients were counted only once for any given event, regardless of the number of times they actually experienced the event. AEs were reported using Medical Dictionary for Regulatory Activities version 25.1. Mono‐R/R and JNJ+VEN: patients with R/R AML who had exhausted or were ineligible for standard therapeutic options, patients with newly transformed secondary AML who exhausted standard therapeutic options during treatment before transformation, and patients with high‐risk or very high risk, R/R MDS who exhausted or were ineligible for standard therapeutic options. The mono‐R/R arm also was to include patients with R/R CMML‐2 who exhausted or were ineligible for standard therapeutic options. JNJ+AZA: patients with newly diagnosed or R/R AML who were unsuitable for intensive treatment with a curative intent (including stem cell transplantation) but eligible to receive AZA, patients with high‐risk or very high‐risk MDS; and patients with CMML‐2. Mono‐LR: patients with very low‐, low‐ or intermediate‐risk MDS and transfusion dependence.

Abbreviations: AE, adverse event; AML, acute myeloid leukaemia; CMML‐2, chronic myelomonocytic leukaemia group 2; JNJ, JNJ‐74856665; LR, lower risk; MDS, myelodysplastic syndromes; R/R, relapsed or refractory; TEAE, treatment‐emergent AE; VEN, venetoclax.

DLTs (Table S8) due to mucositis occurred in 14.3% of patients in the mono‐R/R arm (stomatitis, 13.1%; anal inflammation, 1.2%; and vulvovaginal inflammation, 1.2%). With JNJ+AZA, 11.4% of patients experienced DLTs, most commonly maculopapular rash (5.7%). With JNJ+VEN, 13.8% of patients experienced DLTs, including stomatitis (10.3%). No DLTs occurred in the mono‐LR arm. Mucositis and diarrhoea of any grade were considered AEs of special interest. Overall, 33.3% of patients experienced ≥1 JNJ‐74856665‐related TEAE of mucositis (DLTs, 9.2%), which included anal inflammation, glossitis, mucosal inflammation, pharyngeal inflammation, stomatitis and vulvovaginal inflammation (Table S9). Median (range) mucositis onset across treatment arms was 23 (1–358) days, and most events were Grade 2 (45.1%) or 3 (33.3%). Grade ≥2 mucositis was observed in 60.0%, 30.4% and 13.6% of patients treated with >5, 2.6–5 and <2.6 mg JNJ‐74856665, respectively (Table S10). Overall, 17.6% of patients experienced JNJ‐74856665‐related diarrhoea (no DLTs). Median (range) diarrhoea onset across treatment arms was 35.5 (1–132) days, and most events were Grade ≤2 (88.9%).

ORR for the mono‐R/R arm was 4.8% (4/84; Figure S7). For AML, ORR was 1.5% (1/66; best overall response [bOR] of complete response with incomplete haematological recovery [CRi])—2 (3%) additional patients achieved a bOR of morphologic leukaemia‐free state (MLFS) and 3 (4.5%) achieved a bOR of stable disease (SD). For MDS, ORR was 16.7% (3/18; all bOR of marrow CR [mCR])—3 (16.7%) additional patients had a bOR of SD. In the mono‐LR arm, ORR was 20% (1/5; bOR of mCR)—an additional patient (20%) had a bOR of SD. With JNJ+AZA, ORR was 8.6% (3/35) (AML, 10% [2/20 (both bOR of CRi) plus 2 MLFS and 4 SD]; MDS, 7.1% [1/14 (bOR of mCR) plus 1 SD]; CMML‐2, 0% [0/1]). With JNJ+VEN, ORR was 3.4% (1/29) (AML, 4.2% [1/24 (bOR of CR without minimal residual disease or minimal residual disease‐negative)] plus 1 SD; MDS, 0% [0/5]).

Increased plasma DHO concentrations were observed following JNJ‐74856665 treatment across treatment arms and disease states (Figure S8). Trends towards lower fold change in DHO concentrations were observed with JNJ‐74856665 combination therapy versus monotherapy, and towards higher concentrations of DHO in patients with versus without stomatitis (any grade).

Clinical development of JNJ‐74856665 was driven by single‐agent, preclinical activity showing significant dose‐dependent increase in survival and tumour growth inhibition in xenograft models at doses of 0.062–0.25 mg/kg, and significant increased life span occurred with JNJ‐74856665, JNJ+AZA and JNJ+VEN, and with JNJ+AZA versus AZA or VEN alone. Biological activity of JNJ‐74856665 starting at the lowest administered dose (0.3 mg daily) was confirmed in the phase 1 study. Most frequent toxicity with JNJ‐74856665 was mucositis at all dose levels in a dose‐dependent manner. Planned drug holidays helped reduce incidence and mitigate severity of stomatitis but also may have affected efficacy. Limited clinical activity was observed, and dose escalation was extensively constrained by dose‐dependent toxicity, particularly mucositis, leading to early enrolment stop. In a responder analysis (Supporting Information), responses were seen across treatment arms; however, a common predictor of response was not identified among the limited number of responders, and no additive or synergistic effects of combination therapy were observed clinically. DHO plasma concentrations increased after treatment, providing evidence of target engagement. Whether full, uninterrupted inhibition of DHODH led to improved outcomes in patients with AML and MDS is unknown. While efficacy was limited, observed target engagement and transient limited responses support the potential for clinical activity with DHODHi, as monotherapy or in combination with other agents. Approaches to minimise dose interruptions, mitigate mucositis and identify predictor‐of‐response biomarkers could aid further development of DHODHi in these populations.

## AUTHOR CONTRIBUTIONS

All authors participated in the research. Kathryn Bradford (clinical study), Martin Curtis (clinical study), E. Christine Pietsch (preclinical study), Tammy Bush (preclinical study), Christina D. Guttke (clinical study biomarkers, preclinical) conceived and designed the research study. Emma Searle, Thomas Cluzeau, Jordi Esteve, Emmanuel Gyan, Austin Kulasekararaj, Daniel Morillo, Jenny O'Nions, Arnaud Pigneux, Emmanuel Raffoux, Christian Recher, David Valcárcel, Harriet S. Walter, Kathryn Bradford (clinical study), Adetokunbo Oluwasanjo (clinical study), Martin Curtis (clinical study), Ulrike Philippar (preclinical study), E. Christine Pietsch (preclinical study), Christina D. Guttke (clinical study biomarkers, preclinical), Ana Alfonso Piérola supervised the research. Emma Searle, Thomas Cluzeau, Jordi Esteve, Emmanuel Gyan, Austin Kulasekararaj, Daniel Morillo, Jenny O'Nions, Arnaud Pigneux, Emmanuel Raffoux, Christian Recher, David Valcárcel, Harriet S. Walter and Emmanuel Gyan acquired the data. Kathryn Bradford (clinical study), Adetokunbo Oluwasanjo (clinical study), Martin Curtis (clinical study), Stan Gaj (preclinical study; clinical study biomarker data), E. Christine Pietsch (preclinical study), Xiaochun Zhang (preclinical study), Tammy Bush (preclinical study), Christina D. Guttke (clinical study biomarkers, preclinical) analysed and interpreted the data. Kathryn Bradford (clinical study), Adetokunbo Oluwasanjo (clinical study), Martin Curtis (clinical study), E. Christine Pietsch (preclinical study), Christina D. Guttke (clinical study biomarkers, preclinical), Rachel Pearson (statistician, clinical study) wrote the manuscript, and all authors critically reviewed and/or revised the manuscript.

## CONFLICT OF INTEREST STATEMENT

Emma Searle reports consulting fees from Sanofi, BMS, Johnson & Johnson, Syndax, Cellcentric, Sumitomo, Beigene & Pfizer; meeting and/or travel support from Johnson & Johnson, Abbvie, Beigene, and Menarini Stemline; payment or honoraria from Astellas, Jazz Pharma, Johnson & Johnson, Abbvie & Pfizer; participation on a data safety monitoring board or advisory board for Dark Blue Therapeutics, Sanofi, BMS, Johnson &Johnson, Syndax, Pfizer and Sumitomo; and leadership or fiduciary role (unpaid) with British Society of Haematology Research and Grants (Committee Chair) and ECMC Haematology Network Group (Deputy Chair). Thomas Cluzeau reports consulting fees from Abbvie, Jazz Pharma, Novartis, Agios, Servier, BluePrint and BMS; payment or honoraria from Novartis, Astellas, BMS, Jazz Pharma, Servier, and Incyte; meeting and/or travel support from Pfizer, BMS, Novartis, Abbvie, Servier, Gilead; and participation on a data safety monitoring board or advisory board for BMS, Novartis and KEROS. Jenny O'Nions reports payment or honoraria from Jazz and Astellas; meeting and/or travel support from Janssen, Abbvie, Servier and Jazz; and participation on a data safety monitoring board or advisory board for Abbvie. Christian Recher reports grants or contracts from Abbvie, Astellas, BMS, Jazz Pharma and Iqvia; payment or honoraria from Abbvie, Astellas, BMS, Jazz Pharma, Novartis and Servier; meeting and/or travel support from Abbvie, Astellas, BMS, Jazz Pharma, Novartis and Servier; and participation on a data safety monitoring board or advisory board for Abbvie, Astellas, BMS, Jazz Pharma, Novartis, Servier and Takeda. Emmanuel Gyan reports grants or contracts from Novartis; consulting fees from Janssen, Sanofi, Abbvie and Incyte; payment or honoraria from Gilead and Roche; meeting and/or travel support from Roche; and receipt of equipment, materials, drugs, medical writing, gifts or other services from BMS and Sandoz. Daniel Morillo reports payment or honoraria from Abbvie and GSK; and meeting and/or travel support from Roche, Janssen and Kite. Jordi Esteve reports grants or contracts (to CETLAM cooperative group) from Jazz and Pfizer; payment or honoraria from Abbvie and Jazz; and meeting and/or travel support from Abbvie. Austin Kulasekararaj reports consulting fees from Novartis, SOBI, Alexion/AstraZeneca, Novartis and Roche; payment or honoraria from Celgene/BMS, Novartis, SOBI, Alexion/AstraZeneca, Roche, Samsung, and Amgen; meeting and/or travel support from SOBI and Alexion/AstraZeneca; and participation on a data safety monitoring board or advisory board for Agious, Roche and Biocryst. Emmanuel Raffoux reports consulting fees from Servier and BMS; and payment or honoraria from Servier, BMS and Astellas. Harriet Walter reports research funding paid by Janssen to institutions; medical education grant from Pfizer and research funding from Isogenica and Gilead; payment or honoraria from Abbvie; and participation on a data safety monitoring board or advisory board for Beigene, Eli Lilly and Genmab. Arnaud Pigneux has nothing to report. David Valcárcel reports consulting fees from Amgen, BMS/Celgene, Kite/Gilead, Novartis, SOBI, Astellas, Jazz, MSD and Sanofi; payment or honoraria from Amgen, BMS/Celgene, Grifols, Jazz, MSD, Pfizer, SOBI, Astellas, GEBRO, Janssen, Kite/Gilead, Novartis, Sanofi and Agios; meeting and/or travel support from BMS/Celgene, Jazz, SOBI, Novartis and Sanofi; and participation on a data safety monitoring board or advisory board for Novartis, SOBI, BMS/Celgene, Jazz, Servier, Amgen and Grifols. Nikki Daskalakis, Jacqueline Bussolari, E. Christine Pietsch, Scott Kuduk, Tammy Bush, Xiaochun Zhang, Ulrike Philippar, Adetokunbo Oluwasanjo (at time of study conduct), Kathryn Bradford, Jonathan Miller, Charlotte Van Bogaert, Martin Curtis, Shih‐Yu Chang, Christina Guttke, Stan Gaj, Rachel Pearson, Joseph Murphy and Wim van Dijck are employees of Janssen and may hold company stock in the company. E. Christine Pietsch and Christina Guttke also report patents planned, issued or pending, and Ulrike Philippar also reports a leadership or fiduciary role with J&J Belgium (Board of Directors). Ana Alfonso Piérola reports grants or contracts from AstraZeneca; consulting fees from Jazz Pharmaceuticals and Astellas; payment or honoraria from Novartis, BMS, Jazz Pharmaceuticals, Astellas and Abbvie; and meeting and/or travel support from Kite, BMS and Novartis.

## Supporting information


Appendix S1.

## Data Availability

The data sharing policy of Janssen Pharmaceutical Companies of Johnson & Johnson is available at https://www.janssen.com/clinical‐trials/transparency. As noted on this site, requests for access to study data can be submitted through the Yale Open Data Access (YODA) Project site at http://yoda.yale.edu.
