# Supplementary material for: A phase 1, first‐in‐human, dose‐escalation study of JNJ‐74856665 (dihydroorotate dehydrogenase inhibitor) alone or in combination in patients with AML or MDS
Source: Br J Haematol. 2025 Jul 1;207(2):624–30. doi: 10.1111/bjh.20224 (PMC12379018; doi:10.1111/bjh.20224)
Supplement: Supplementary file 1 — Appendix S1. [file BJH-207-624-s001.pdf]

SUPPORTING INFORMATION

A phase 1, first-in-human, dose-escalation study of JNJ-74856665 (dihydroorotate dehydrogenase inhibitor) alone or in combination in patients with AML or MDS

Emma Searle, Thomas Cluzeau, Jordi Esteve, Emmanuel Gyan, Austin Kulasekararaj, Daniel Morillo, Jenny O'Nions, Arnaud Pigneux, Emmanuel Raffoux, Christian Recher, David Valcárcel, Harriet S. Walter, Nikki Daskalakis, Jacqueline Bussolari, E. Christine Pietsch, Scott Kuduk, Tammy Bush, Xiaochun Zhang, Ulrike Philippar, Adetokunbo Oluwasanjo, Kathryn Bradford, Jonathan Miller, Charlotte Van Bogaert, Martin Curtis, Shih-Yu Chang, Christina Guttke, Stan Gaj, Rachel Pearson, Joseph Murphy, Wim van Dijck, and Ana Alfonso Piérola

Abstract

Dihydroorotate dehydrogenase (DHODH) is a key enzyme in *de novo* pyrimidine synthesis and represents a metabolic target in acute myeloid leukaemia (AML) and myelodysplastic syndromes (MDS). Preclinical *in vivo* studies demonstrated induction of differentiation after treatment with JNJ-74856665, an oral and selective inhibitor of DHODH, which supported further clinical study. In this phase 1 dose-escalation study, the safety and efficacy of JNJ-74856665 were evaluated as monotherapy and in combination with azacitidine (AZA) or venetoclax (VEN) in patients with newly diagnosed or relapsed/refractory (R/R) AML, R/R MDS, or R/R chronic myelomonocytic leukaemia-2 (CMML-2) (NCT04609826). In total, 153 patients were enrolled into 1 of 2 JNJ-74856665 monotherapy arms (mono-R/R or mono-LR [only lower risk MDS]) or 1 of 2 combination therapy arms (JNJ+AZA or JNJ+VEN). Overall response rates for AML and MDS across all dose cohorts were 1.5% (1/66) and 16.7% (3/18) in the mono-R/R arm; 10% (2/20) and 7.1% (1/14) in the JNJ+AZA arm; and 4.2% (1/24) and 0% (0/6) in the JNJ+VEN arm, respectively. Overall response rate for MDS in the mono-LR arm was 20% (1/5). Across all treatment arms, the most common JNJ-74856665-related toxicity was stomatitis, which prevented further dose escalation and resulted in halting enrolment before dose expansion.

Preclinical studies: Methods

Human AML cell line studies

MOLM-13, HL-60, OCI-AML3, and THP-1 cell lines (Deutsche Sammlung von Mikroorganismen und Zellkulturen) were cultured at 37°C, 5% CO_2 in the recommended medium. To assess the anti-proliferative activity of JNJ-74856665, MOLM-13, HL-60, OCI-AML3, and THP-1 cells were seeded either in 96-well plates (in triplicate) and exposed to JNJ-74856665 using a 9-point, 6-fold serial dilution (range, 3 µM–3 pM) or in 384-well plates (in duplicate) and exposed to JNJ-74856665 using an 11-point, 3-fold serial dilution (range, 100 nM–1.7 pM). Half of the wells were incubated with JNJ-74856665, and half were incubated with JNJ-74856665 + 100 µM uridine (control for on-target activity). After 72 hours of incubation at 37°C and 5% CO_2, cell viability was determined by adding CellTiter-Glo. Luminescence was read on an Envision device (PerkinElmer) for 96-well plates, or a PHERAstar device (BMG LABTECH) for 384-well plates, and readouts were used to calculate the IC_{50} values. Collected luminescence values were used to calculate JNJ-74856665 activity using Prism (GraphPad, version 7.0; 96-well plates) or Genedata Screener (Genedata, version 16.0, 384-well plates). Log_{10} JNJ-74856665 concentrations versus percent changes in cell number relative to negative control were plotted, and data was fitted to a 4-parameter logistic regression to determine IC_{50} values (ie, inhibitory concentration causing 50% change in cell number relative to control).

Effects of JNJ-74856665 on differentiation marker mRNA expression were assessed in MOLM-13, HL-60, OCI-AML3, and THP-1 cells using QuantiGene Plex (QGP) technology. Cells were plated (MOLM-13, HL-60, THP-1: 60,000 cells/well or OCI-AML3: 30,000 cells/well) either in 96-well plates (in triplicates) in the presence of increasing JNJ-74856665 concentrations or 3 and 30 nM of JNJ-74856665 ± 100 µM uridine. After incubation (MOLM-13, 24 hours; HL-60, 48 hours; OCI-AML3, 24 hours; or THP-1, 72 hours), cells were lysed using the sample processing kit and incubated for 30 minutes at 55°C. Cell lysates were mixed with target-specific capture probes, Luminex beads, and blocking probes; transferred to the custom assay hybridization plate; and incubated for 18–22 hours at 54°C. Plates were transferred to a magnetic separation plate and washed with 1 × QGP wash buffer to remove unbound materials from beads, followed by sequential hybridization of preamplifiers, amplifiers, and label probe, as well as streptavidin R-phycoerythrin binding. The resulting fluorescence signal associated with individual capture beads was read on a FlexMap 3-dimensional flow cytometer

(Luminex). Signal was reported as median fluorescence intensity (MFI), and MFI was acquired for the housekeeping gene, HPRT1, and the cluster of differentiation markers CD11b and CD14. MFI for each differentiation gene was normalized to the MFI of the housekeeping gene. Data was analysed using Excel (Microsoft) and Prism (version 7.0).

Effects of JNJ-74856665 on cell cycle arrest were assessed in MOLM-13, HL-60, and OCI-AML3 cells. Cells were seeded in 6-well plates at 3.0×10^5 cells/mL (1 well/treatment) and exposed to 0, 0.1, 1, or 10 nM of JNJ-74856665 ± 100 μM uridine for 24 hours. Cells then were harvested, fixed, and permeabilized in 70% ethanol. Cells were washed twice, first with phosphate-buffered saline and then in stain buffer, to remove the ethanol. Washed cells were stained with 0.25 mL/well of propidium iodide with ribonuclease (15 minutes, 25°C). Data was acquired on a Celesta flow cytometer (BD Biosciences) and analysed for cell cycle stages.

Effects of JNJ-74856665 on the induction of apoptosis were assessed in MOLM-13 and OCI-AML3 cells. Cells were seeded (in triplicate) in 96-well plates at 1.5×10^5 cells/m, and HL-60 and cells were seeded (in triplicate) in 96-well plates at 3.0×10^5 cells/mL. Cells were grown for 72 hours in the presence of 3.3 and 30 nM JNJ-74856665 in the presence and absence of 100 μM uridine for 72 hours. Cells were pelleted and washed twice with cold stain buffer, then resuspended in 50 μL of Annexin V binding buffer at a concentration of $0.25–1.0 \times 10^6$ cells/mL, followed by 1 μL of Annexin V–allophycocyanin and 2 μL of 7-aminoactinomycin D (7-AAD). After 15 minutes of incubation at room temperature, protected from light, 200 μL of Annexin V binding buffer was added to each well. Subsequently, cells were acquired on a Celesta flow cytometer to determine the percent of live (Annexin V^-/7-AAD^-), early (Annexin V^+/ 7-AAD^-), and late (Annexin V^+/7-AAD^+) apoptotic cells.

Animal studies

Female NSG (ie, non-obese diabetic severe combined immunodeficiency gamma or non-obese diabetic Cg-*Prkdc*^{scid} *Il2rg*^{tm1Wjl}*/SzJ*) mice were used when they were approximately 6–8 weeks old and weighed approximately 25 g. The mice were allowed to acclimate and recover from shipping-related stress for ≥5 days before experimental use.

For SC tumour models, each female NSG mouse received 2×10^6 MOLM-13 or 1×10^6 OCI-AML3 cells (total volume: 0.2 mL) in the right flank. For the MOLM-13 disseminated AML xenograft model (survival assessments), each female NSG mouse received 1×10^5 cells via intravenous injection (total volume: 0.2 mL). JNJ-74856665 was formulated in 0.5% hydroxypropyl methylcellulose, and

vehicle or JNJ-74856665 were orally administered daily. Animal studies were carried out in accordance with The Guide for the Care and Use of Laboratory Animals and were approved by the Institutional Animal Care and Use Committee of Janssen R&D, Spring House, PA.

For pharmacodynamic analysis, DHO levels were measured in plasma, and DHO and differentiation marker induction were assessed in subcutaneous MOLM-13 tumours isolated from mice collected 24 hours after the last dose of a 10-day, oral, daily JNJ-74856665 administration regimen. Plasma samples were processed using a protein precipitation method: a 10-μL aliquot was pipetted into the wells of a 2-mL, 96-well, protein precipitation polypropylene filter plate, which was attached on top of a 96-well sample injecting plate. Then, 100 μL of internal-standard spiking solution containing 250 nM $^{13}C_{11}$-tryptophan and 1% formic acid in pure acetonitrile was added to each sample. The 2-plate assembly was vortexed on a plate shaker (Eppendorf) at a setting of 1200 rpm for 0.5 minutes, and then centrifuged at 4°C in an Eppendorf 5804R (Eppendorf) at 1600 g for 3 minutes. The protein precipitation filter plate was removed, and the sample-injecting plate was capped and placed into an SIL-30ACMP autosampler (Shimadzu) set at 10°C. The injection volume was 10 μL. Tumour samples for DHO analysis were transferred into pre-chilled (liquid nitrogen) ceramic mortars and merged with approximately 5 mL of liquid nitrogen. Using a pre-chilled pestle in liquid nitrogen, the tumour piece was ground into fine powder. The weight of homogenized tissue powder was recorded for normalization of DHO concentrations. Then, 300 μL of pre-chilled (wet ice) methanol:water solution (2:98 [volume/volume]) with 1% formic acid was added to each tube containing tumour powder. The tubes were vortexed for 30 seconds on a Lab Dancer (VWR). The tumour homogenate was further purified using the protein precipitation method described above. The remaining tumour homogenate was stored at -80°C until analysed.

Processed plasma and tumour samples were analysed for DHO levels by liquid chromatography–tandem mass spectrometry, with $^{13}C_{11}$-tryptophan used as the internal standard. The high-performance liquid chromatography system was interfaced with an API 5000™ mass spectrometer (Applied Biosystem) operated in negative electrospray mode. The multiple reaction monitoring transitions for DHO and the internal standard were 157.0→70.0 and 214.0→124.0, respectively. Data acquisition and processing were carried out by Analyst software (version 1.7; Applied Biosystems). Calibration curves were constructed by plotting peak area ratio of DHO to internal standard against analyte concentrations. Concentrations of calibration curve of DHO were 5, 10, 25, 100, 500, 1000, 1600, and 2000 ng/mL. Quality control samples contained DHO concentrations at 15, 100, 800, and 1500 ng/mL and were included at the

beginning and the end of each analytical run. Linear regression with weighing factor of 1/×2 was used. The correct weighting factor is determined by the relationship between the standard deviation of instrument responses (ie, σ) and the concentrations of analytes (ie, x). The weighting factor of 1/×2 was selected because σ was proportional to x in this method. Assessment of differentiation marker induction in MOLM-13 tumours was done by QuantiGene Plex technology. Frozen tumours were homogenized in individual lysing matrix tubes with working homogenizing solution using a FastPrep-24 (MP Biomedicals) and lysates were processed as described above.

The ability of treatment with JNJ-74856665, alone or in combination with AZA or VEN, to produce a survival advantage—as measured by increased life span (ILS)—was evaluated in the MOLM-13 disseminated AML xenograft model. JNJ-74856665 doses were selected based on the aforementioned monotherapy efficacy studies using the MOLM-13 model. Each free-base compound was prepared to deliver a total volume of 0.16 mL intraperitoneal (8 mL/kg) per dose for a 20-g mouse. JNJ-74856665 was formulated in 0.5% hydroxypropyl methylcellulose and working stocks were prepared once per week and stored at 4°C. AZA was formulated in 0.9% saline and working stock was prepared daily and stored at 25°C. VEN was formulated in 10% ethanol/30% polyethylene glycol 400 and working stock was prepared daily and stored at 25°C. Doses of each drug were adjusted by individual body weight each day.

Female non-obese diabetic severe combined immunodeficiency gamma or non-obese diabetic Cg-$\textit{Prkdc}^{scid}$ $\textit{Il2rg}^{tm1Wjl}$/SzJ mice received 1 × 10^5 MOLM13 cells intravenously in a total volume of 0.1 mL phosphate-buffered saline. Mice were randomized by body weight (average, approximately 22.5 g/group) and assigned to treatment groups on day 6 post tumour implantation (12/group). Treatment with JNJ74856665 0.063 mg/kg, VEN 100 mg/kg, or appropriate vehicle was initiated on day 6, with daily oral dosing for 42 days. Treatment with AZA 2 mg/kg or saline vehicle also was initiated on day 6, with daily intraperitoneal dosing using the schedule 7 days on/14 days off/6 days on. VEN and AZA were administered in the morning, whereas JNJ74856665 was dosed 6–8 hours later. The vehicle control group was dosed with each vehicle according to the same schedule and route as their respective drugs. For survival assessment, results were plotted as the percent survival against days post-tumour implant. Negative clinical signs, such as hind limb paralysis, poor body condition, and/or ≥20% body weight loss, were used as surrogate endpoints for death and animals were removed from study. Median survival was determined using Kaplan-Meier survival analysis. Percent ILS was calculated using the formula:

($[MS_t MS_c]/MS_c$) × 100, where MS_t was the median survival of the treatment group and MS_c was the median survival of the control group. As defined by National Cancer Institute criteria, ≥25% ILS was considered biologically significant.

Statistical significance was evaluated for Kaplan-Meier survival plots, comparing each therapeutic treatment group with the vehicle control group or comparing treatment groups to each other, using the log-rank (Mantel-Cox) test in R software version 3.4.2 (using Janssen's internally developed Shiny application version 4.0, Survival application). For all analyses, differences between groups were considered significant when $p \leq 0.05$.

AML patient sample studies

JNJ-74856665 was tested alone or in combination with azacitidine (AZA) or venetoclax (VEN) in proliferation assays using 30 primary, leukapheresis-derived, AML patient samples (Deutsche Sammlung von Mikroorganismen und Zellkulturen). The CellTiter-Glo™ assay was implemented in a 96-well plate format, testing different combinations of drug treatments (**table below**). AML samples were cultured in complete medium and seeded at a density of 20,000 cells/100 µL per well. Drugs were added at 2-times the required concentration in 100 µL of serum-free expansion medium on the same day. Plates were cultured for 6 days at 37°C, 5% CO_2, with no further additions or changes to the medium. ATP levels, used as a surrogate marker of viability, were evaluated on day 6. Briefly, plates were removed from the incubator and allowed to equilibrate at room temperature up to 30 minutes. CellTiter-Glo was added (100 µL/well) and mixed on a plate rocker for 2 minutes. Plates were subsequently incubated for a further 10 minutes at room temperature to stabilize the luminescent signal before quantitation at 485–500 nm excitation/520–530 nm emission using a plate reader (Tecan). Half maximal inhibitory concentration (IC_{50}) values were determined in Prism, CalcuSyn (Biosoft, Cambridge, UK), and the monotherapy fit of the biochemically intuitive generalized Loewe (BIGL) package.[1] Synergy evaluations were performed using the BIGL package.

Drug concentrations used in viability assays of AML patient samples

JNJ-74856665 (nM)	Venetoclax (nM)	Azacitidine (nM)	JNJ-74856665 (nM) + venetoclax (nM)	JNJ-74856665 (nM) + azacitidine (nM)
3000	500	10 000	1000 + 500	1000 + 10,000
1000	50	1 000	100 + 500	100 + 1000
100	5	100	10 + 5	10 + 100
10	0.5	10	1 + 0.5	1 + 10
1	0.05	1	0.1 + 0.05	0.1 + 1
0.1	0.005	0.1	0.01 + 0.005	0.01 + 0.1

AML, acute myeloid leukaemia.

Preclinical studies: Results

JNJ-74856665 induced anti-proliferative activity against AML cell lines (**table below**).

Anti-proliferative activity of JNJ-74856665 against a panel of AML cell lines.

Cell line	IC_{50} (nM) JNJ-74856665	IC_{50} (nM) JNJ-74856665 + 100 μM uridine
MOLM-13	0.2 ± 0.1 (*n* = 11)	>3,000 (*n* = 3)
HL-60	0.3 ± 0.2 (*n* = 7)	>3,000 (*n* = 7)
OCI-AML3	0.6 ± 0.2 (*n* = 6)	>3,000 (*n* = 6)
THP-1	0.6 ± 0.3 (*n* = 10)	>3,000 (*n* = 3)

AML, acute myeloid leukaemia; IC_{50}, 50% inhibitory concentration; n, number of experiments.

JNJ-74856665 induced mRNA expression of differentiation markers (**figure below**) in a concentration-dependent manner (**A**), which was accompanied by cell cycle arrest in S phase (**B**) and induction of apoptosis (**C**). Co-treatment of AML cell lines with JNJ-74856665 and uridine rescued the antiproliferative, differentiation-promoting, cell-cycle–inhibitory, and pro-apoptotic effects of DHODH inhibition by JNJ-74856665.

Induction of differentiation marker mRNA expression (A) on cell cycle arrest (B) and apoptosis induction (C) in AML cell lines treated with JNJ-74856665 ± uridine.

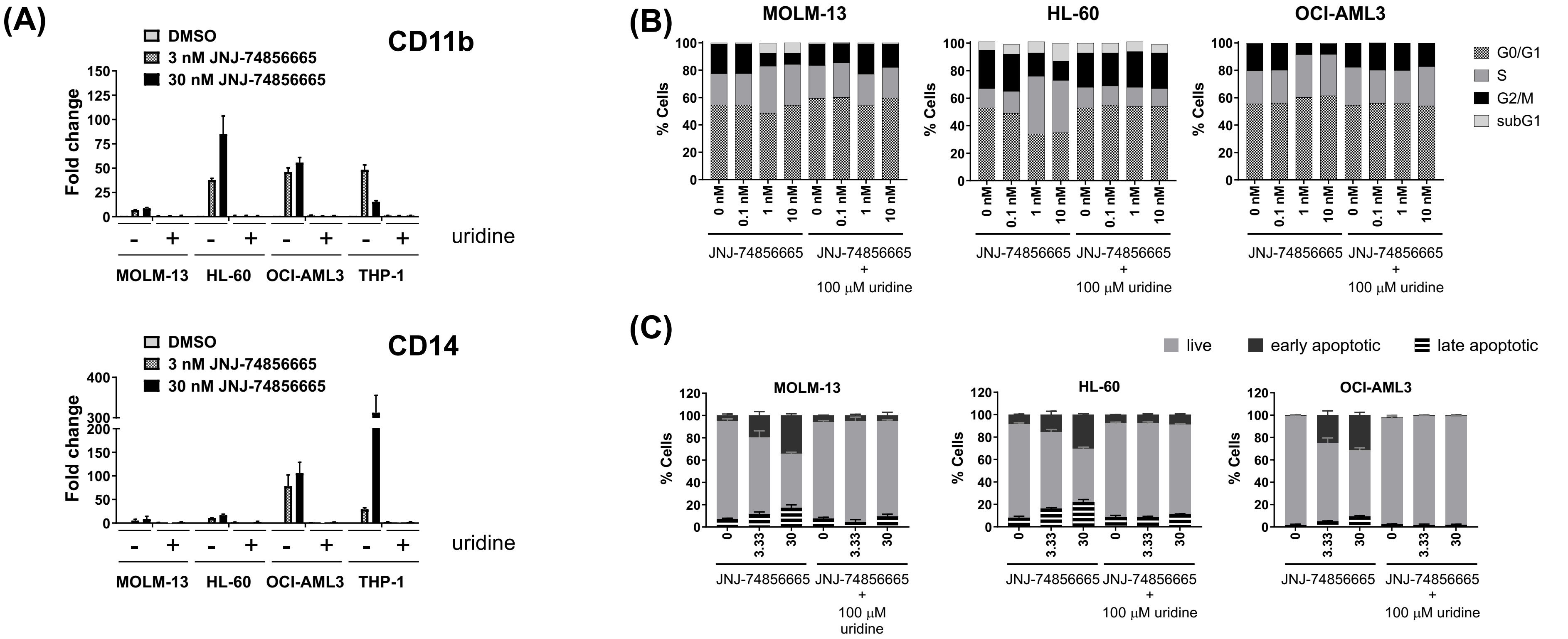

AML, acute myeloid leukaemia.

In vivo (**figure below**), JNJ-74856665 induced decreases in leukaemic burden in SC MOLM-13 (**A**) and SC OCI-AML3 (**B**) tumour models, and increased survival in the MOLM-13 disseminated AML xenograft model (**C**). In pharmacodynamic investigations of SC MOLM-13 tumours isolated after 10 days of daily JNJ-74856665, DHO was upregulated (**D**) and differentiation markers were induced (**E**), demonstrating target engagement.

Effect of JNJ-74856665 on leukaemic burden in MOLM-13 subcutaneous tumours (A) and OCI-AML3 subcutaneous tumours (B), survival of mice bearing MOLM-13 disseminated tumours (C), pharmacodynamic markers (D), and differentiation marker induction (E).

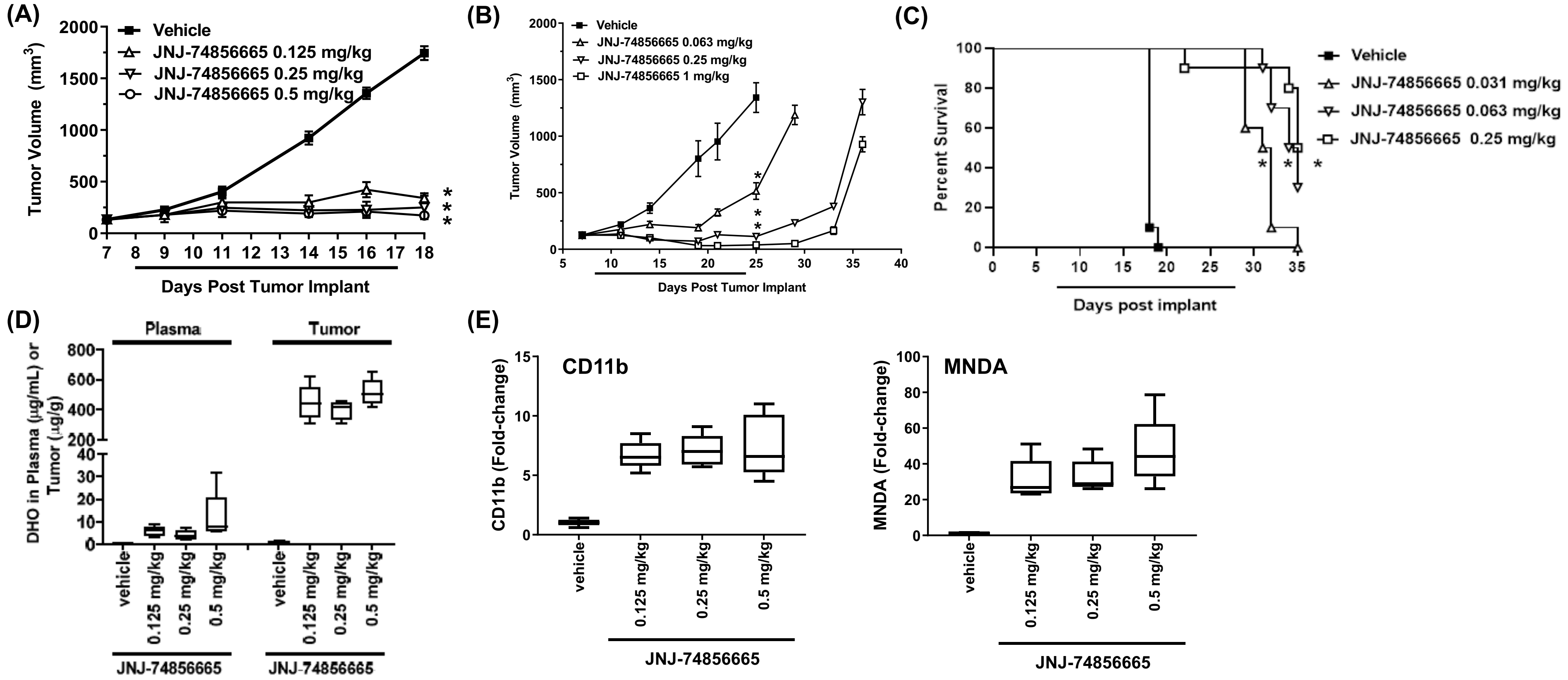

CD, cluster of differentiation; DHO, dihydroorotate; MNDA, myeloid cell nuclear differentiation antigen; SC, subcutaneously; SEM, standard error of the mean; TGI, tumour growth inhibition.

A and B: Group tumour volumes are graphed as mean ± SEM (n = 10 per group). MOLM-13 cells were implanted SC on day 0, and mice bearing established tumours were dosed orally with vehicle or JNJ-74856665 once daily on days 8–17 (as denoted by the horizontal bar below the X-axis). Administration of 0.125, 0.25, and 0.5 mg/kg JNJ-74856665 resulted in significant change in TGI of 87%, 93%, or 97%, respectively, compared to the vehicle control (A). OCI-AML-3 cells were implanted SC on day 0, and mice bearing established OCI-AML3 tumours were dosed orally with vehicle or JNJ-74856665 once daily on day 8–24 (as denoted by the horizontal bar below the X-axis). Administration of 0.063, 0.25 and 1 mg/kg JNJ-74856665 resulted in significant change in TGI of 68%, 101%, or 107%, respectively, compared to the vehicle-treated control group (B).

C: Molm-13 tumour cells were implanted IV on day 0, and mice were dosed orally with vehicle or JNJ-74856665 once daily on days 7–27 (as denoted by the horizontal bar below the X-axis). Data is displayed to day 35, when all mice were removed from study (n = 10 per group). JNJ-74856665 at 0.031, 0.063, or 0.25 mg/kg dosed orally daily for 21 days significantly increased the life span of tumour-bearing mice by 75%, 92%, or 94%, respectively, compared to the vehicle control group. *Denotes significant difference ($p \le 0.05$) versus vehicle control (A-C).

D and E: Mice bearing MOLM-13 tumours (shown in A) were evaluated for PD effects after the 10th daily dose of JNJ-74856665. In response to continuous dosing, marked increases in DHO were observed in MOLM-13 tumours and plasma, suggesting sustained target engagement (D). Administration of JNJ-74856665 for 10 days mediated robust induction of *CD11b* (6–7 fold) and *MNDA* (30–45 fold) differentiation marker mRNA expression over vehicle control tumours (E).

In an *in vitro* viability assay, a potent cytotoxic response (half maximal inhibitory concentration [IC_{50}] values ≤32 nM) was observed in 16/30 (53.3%) AML patient samples exposed to JNJ-74856665. IC_{50} values in remaining samples were 211 nM (n = 1), 466 nM (n = 1), and >3000 nM (n = 12), the highest concentration tested. JNJ-74856665+AZA and JNJ-74856665+VEN demonstrated lack of antagonism (data not shown).

Compared with vehicle (median survival, 19 days), mice bearing disseminated MOLM-13 tumours had significantly increased life span (ILS) with single-agent JNJ-74856665 (90% ILS; median survival, 36 days [$p \le 0.05$]) or AZA (37% ILS; 26 days [$p \le 0.05$]) (**figure below**). Furthermore, JNJ-74856665+AZA significantly increased ILS (158%; 49 days [$p \le 0.05$]) versus single-agent treatments.

Median survival was alike with vehicle and VEN alone (19 days). JNJ-74856665+VEN provided no added survival benefit versus single-agent JNJ-74856665 (37 vs 36 days), and JNJ-74856665+AZA+VEN did not provide ILS versus JNJ-74856665+AZA (**figure below**).

Effect of treatment with JNJ-74856665, AZA, VEN, JNJ-74856665+AZA, JNJ-74856665+VEN, JNJ-74856665+AZA+VEN, and AZA+VEN on survival of female non-obese diabetic severe combined immunodeficiency gamma or non-obese diabetic Cg-Prkdc^{scid} $\mathit{Il2rg}^{tm1Wjl}$/SzJ mice bearing MOLM-13 disseminated tumours.

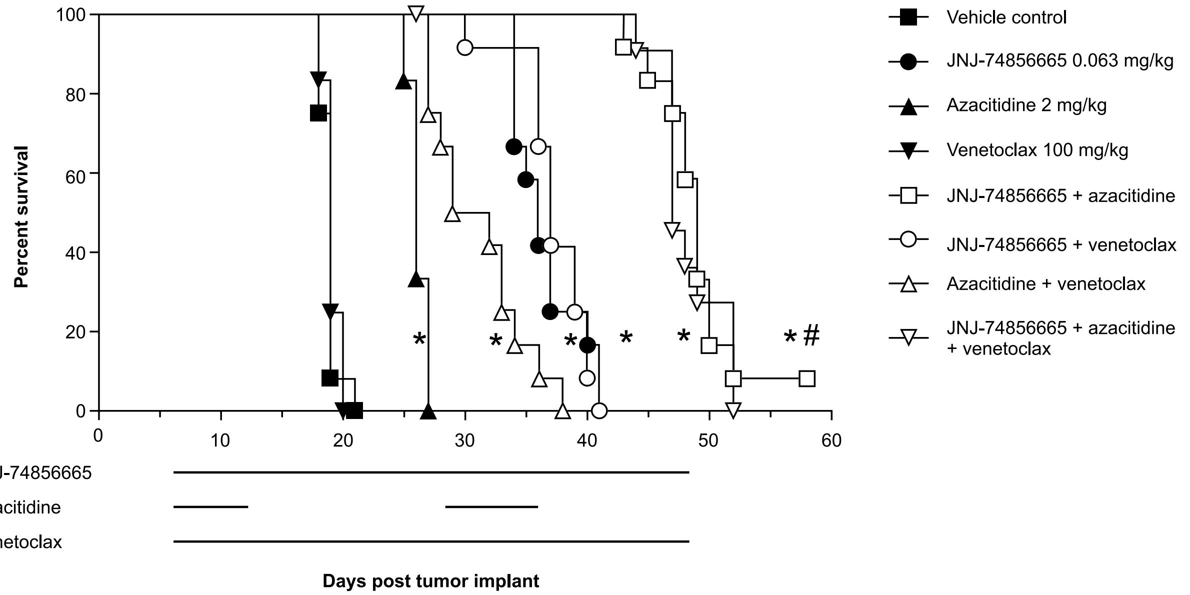

MOLM-13 tumour cells were engrafted intravenously on day 0, and treatments started on day 6 (12 mice/group). Lines underneath the X-axis indicate the dosing period for each drug. JNJ-74856665 and VEN were orally dosed daily on days 6–47 for a total of 42 doses; AZA was dosed intraperitoneally daily on days 6–12 and 27–33, for a total of 14 doses. Dose levels for drugs in the combination groups were the same as for the respective monotherapy groups. Mice in the vehicle control group were dosed with vehicles for JNJ-74856665, AZA, and VEN on the appropriate days. Data are displayed to day 58, when all remaining mice were removed from study.

* Denotes significant difference ($p \leq 0.05$) from vehicle control group.

[#] Denotes significant difference ($p \leq 0.05$) of JNJ-74856665+AZA group from JNJ-74856665 or AZA monotherapy groups.

Clinical study: Methods

Design and execution

The clinical study (**Figure S1**) was conducted at 20 centers in France, Republic of Korea, Spain, and the United Kingdom. Enrolment occurred from December 1, 2020 to the data cut-off of August 1, 2022. In addition to the primary objectives described in the main Methods section, secondary objectives included assessment of the pharmacokinetics, pharmacodynamics, and preliminary clinical activity of JNJ-74856665 as monotherapy and in combination with AZA or VEN.

Trial participation was to include 3 periods: a screening phase, a treatment phase, and a post-treatment follow-up phase. The treatment phase—required for all patients, including those who discontinue study treatment for any reason, except for lost to follow-up, death, or withdrawal of consent—began with the first dose of study treatment and continued until the end-of-treatment visit, which was completed ≤30 days after the last study treatment dose and before starting the first subsequent anticancer therapy. The post-treatment follow-up phase started after the end-of-treatment visit and continued until the patient completed or withdrew from the study. The dose-limiting–toxicity (DLT) evaluation period—for dose escalation and expansion—was considered the first 21 (JNJ-74856665 monotherapy [mono-R/R and mono-LR]) or 28 (JNJ-74856665 combination therapy [JNJ+AZA and JNJ+VEN]) days after initial JNJ-74856665 dosing. Adverse events (AEs) were evaluated using National Cancer Institute Common Terminology Criteria for AE (version 5.0). AEs of special interest (AESI) of any grade included mucositis and diarrhoea.

Fig. S1: Clinical study design.

Mono-R/R: JNJ-74856665 monotherapy in patients with r/r AML, MDS, or CMML-2,[a] or
Mono-LR: JNJ-74856665 monotherapy in patients with lower risk MDS

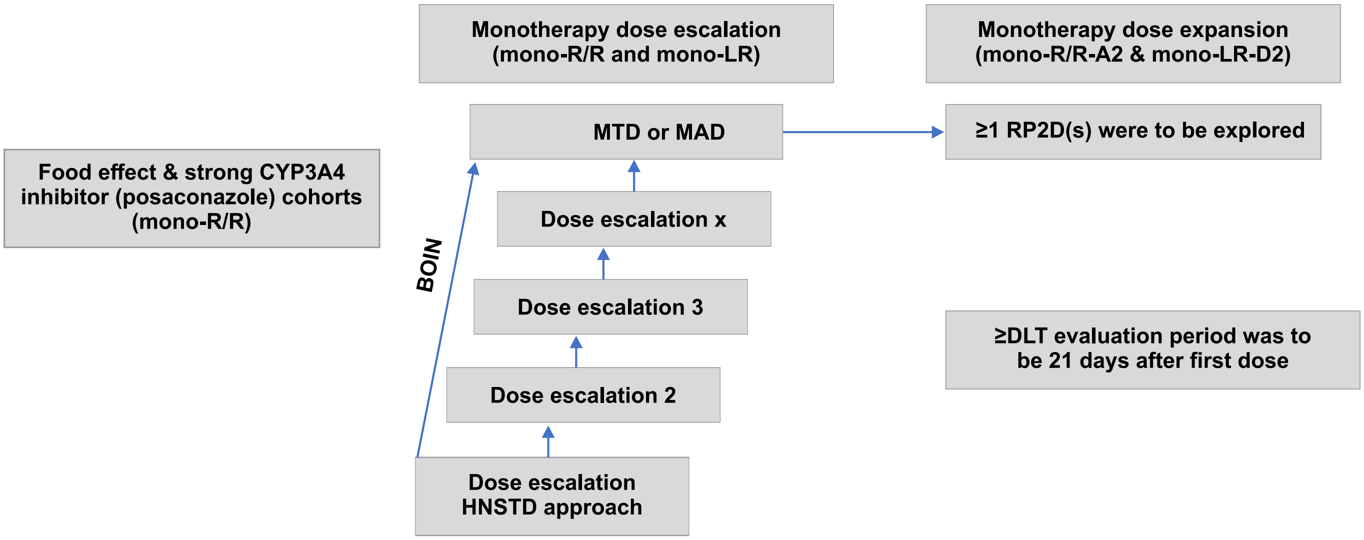

JNJ+AZA: JNJ-74856665+azacitidine in patients with newly diagnosed or r/r AML, MDS, or CMML-2

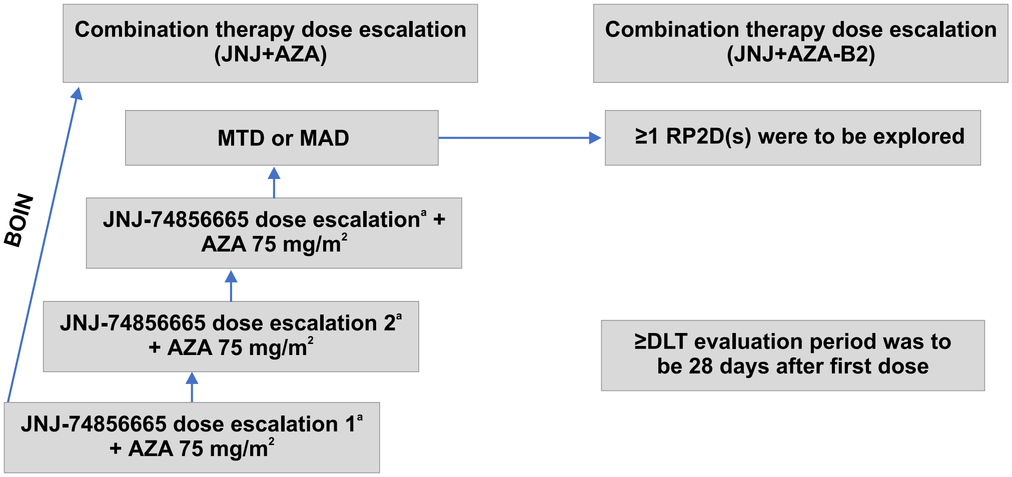

JNJ+VEN: JNJ-74856665+venetoclax in patients with newly diagnosed or r/r AML, MDS, or CMML-2

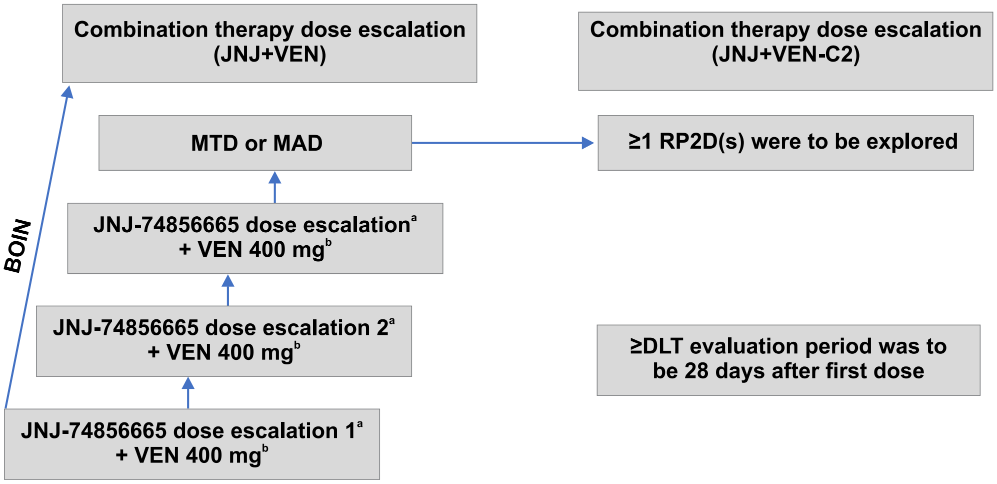

AML, acute myeloid leukaemia; AZA, azacitidine; BOIN, Bayesian optimal interval; CMML-2, chronic myelomonocytic leukaemia group 2; DLT, dose-limiting toxicity; HNSTD, highest non-severely toxic dose; JNJ, JNJ-74856665; LR, lower risk; MAD, maximal administered dose, MDS, myelodysplastic syndromes; MTD, maximal tolerated dose(s); RP2D, recommended phase 2 dose; R/R, relapsed or refractory; SET, study evaluation team; VEN, venetoclax.

Mono-R/R and JNJ+VEN: patients with R/R AML who had exhausted or were ineligible for standard therapeutic options, patients with newly transformed secondary AML who exhausted standard therapeutic options during treatment before transformation, and patients with high-risk or very high-risk, R/R MDS who exhausted or were ineligible for standard therapeutic options. The mono-R/R arm also was to include patients with R/R CMML-2 who exhausted or were ineligible for standard therapeutic options.

JNJ+AZA: patients with newly diagnosed or R/R AML who were unsuitable for intensive treatment with a curative intent (including stem cell transplantation) but eligible to receive AZA, patients with high-risk or very high-risk MDS; and patients with CMML-2.

Mono-LR: patients with very-low, low, or intermediate-risk MDS and transfusion dependence.

[a] JNJ-74856665 dose escalation in the combination arms began with a dose from the Mono-R/R determined as safe by the SET and may have begun after ≥2 dose levels were completed in mono-R/R and before achieving the MTD(s)/RP2D JNJ-74856665 monotherapy dose in mono-R/R; each combination arm-dose level was at or below the mono-R/R dose level considered safe by the SET.

[b] The full VEN dose may have been adjusted for individual patients based on comedication with CYP3A4-inhibiting medication(s).

Dosing

Oral JNJ-74856665 dosing started at 0.3 mg daily (**Figure S1**). Intravenous or subcutaneous AZA 75 mg/m^2/day was administered for 7 days every 4 weeks and, following a 3-day ramp up, oral VEN 400 mg was administered daily. Patients received study treatments until progressive disease (PD), unacceptable toxicity, or other treatment discontinuation criteria were met. In mono-R/R, dose escalation started at 0.3 mg; other arms were initiated after ≥2 dose levels were completed in mono-R/R. In all arms, further dose escalation was guided at each dose level by Bayesian optimal interval (BOIN) design (**figure and table below**) based on the number of patients treated within a dose cohort and the number of DLTs observed, as well as all available safety, pharmacokinetic, pharmacodynamics, biomarker, or preliminary clinical activity data. If a dose escalation cohort enrolled >1 patient, a staggered dose strategy between patients was applied, with a minimal interval of 3 days between the first dose of the first patient and subsequent patients. Subsequent

dose levels were approved by the study evaluation team. At each step, a new JNJ-74856665 dose cohort could not exceed 3 times the previous dose level in the mono-R/R and mono-LR arms, and 2 times the previous dose level in the JNJ+AZA and JNJ+VEN arms. Thus, the maximal rate of dose escalation in combination arms could not exceed that of the mono-R/R arm. JNJ-74856665 dose levels in combination arms were at or below the highest dose determined to be safe by the study evaluation team in the mono-R/R arm.

Dose escalation flowchart based on BOIN design.

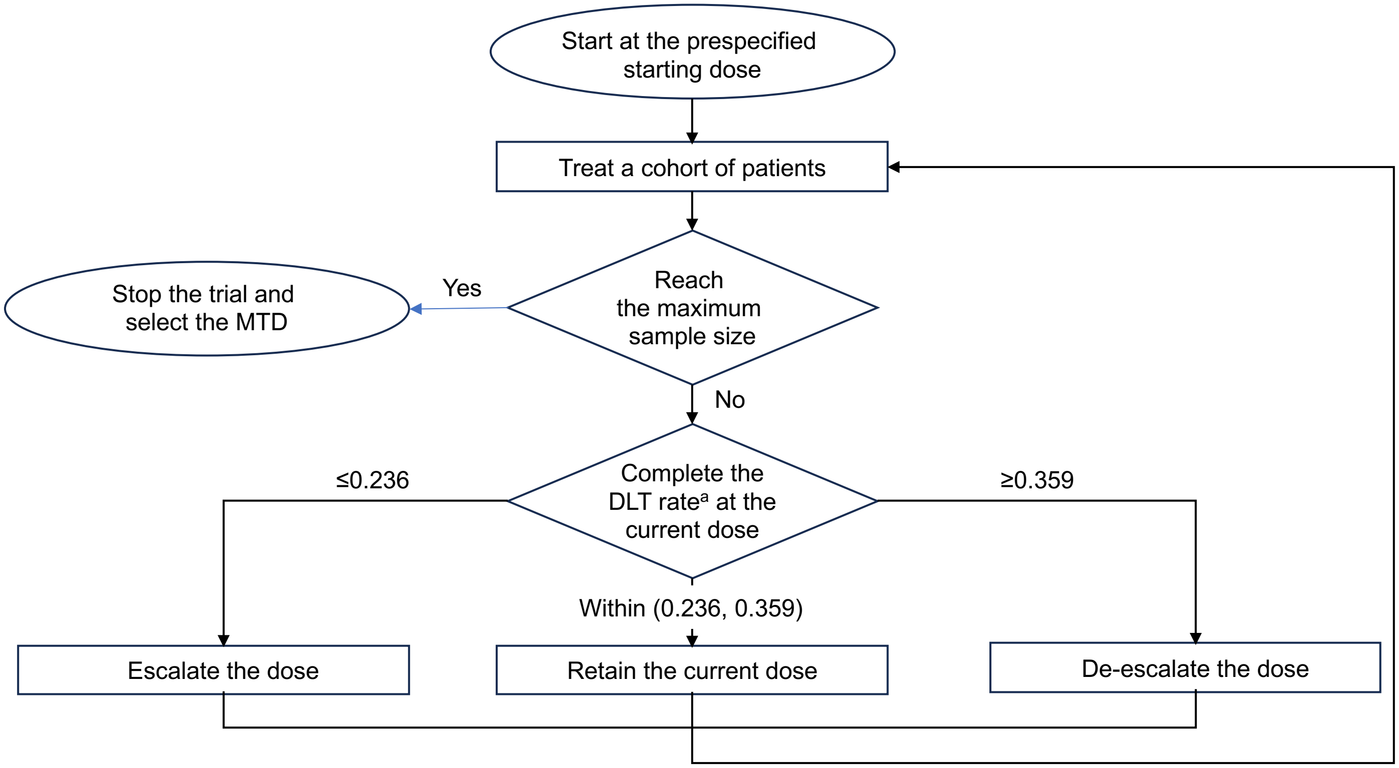

^{a}DLT rate = $\frac{\text{Total number of patients who experienced DLT at the current dose}}{\text{Total number of DLT-evaluable patients treated at the current dose}}$

BOIN, Bayesian optimal interval; DLT, dose-limiting toxicity; MTD, maximum tolerated dose.

Dose level recommendations based on BOIN design.

	Patients treated at a current dose level															
**Dose level action**	**1**	**2**	**3**	**4**	**5**	**6**	**7**	**8**	**9**	**10**	**15**	**20**	**25**	**30**	**35**	**30**
Escalate if number of patients who experienced DLT was ≤	0	0	0	0	1	1	1	1	2	2	3	4	5	7	8	9
Hold if number of patients who experienced DLT was =	NA	NA	1	1	NA	2	2	2	3	3	4-5	5-7	6-8	8-10	9-12	10-14
De-escalate if number of patients who experienced DLT was ≥	1	1	2	2	2	3	3	3	4	4	6	8	9	11	13	15
Eliminate if number of patients who experienced DLT was ≥	NA	NA	3	3	4	4	5	5	5	6	8	10	12	14	15	17

BOIN, Bayesian optimal interval; DLT, dose-limiting toxicity; NA, not applicable.

Patients

Table S1: Clinical study inclusion and exclusion criteria.

	Mono-R/R	JNJ+AZA	JNJ+VEN	Mono-LR
**Inclusion criteria**				
Age	≥18 years			
Diagnosis	• AML according to WHO 2016 criteria[2] with R/R disease and have exhausted or are ineligible for standard therapeutic options; or	Considered unsuitable for intensive treatment with a curative intent, including stem cell transplantation, but eligible to receive	• AML according to WHO 2016 criteria[2] with R/R disease and have exhausted or are ineligible for standard therapeutic options; or	Very low, low, or intermediate-risk MDS according to WHO 2016 criteria[2] and IPSS-R[3] and<br>• Transfusion dependence: requiring

	• Newly transformed secondary AML according to WHO 2016 criteria[2] and have exhausted standard therapeutic options for AML during treatment prior to transformation; or<br>• High-risk or very high-risk MDS according to WHO 2016 criteria[2] and the revised IPSS-R[3] with R/R disease and have exhausted or are ineligible for standard therapeutic options; or<br>• CMML-2 according to WHO 2016 criteria[2] with R/R disease and have exhausted or are ineligible for standard therapeutic options	AZA with the following underlying diseases:<br>• AML (newly diagnosed or R/R) according to WHO 2016 criteria[2] only if venetoclax + hypomethylating agent (or low-dose cytarabine) is not indicated or available; or<br>• High-risk or very high-risk MDS according to WHO 2016 criteria[2] and IPSS-R[3]; or<br>• CMML-2 according to the WHO 2016 criteria[2]	• Newly transformed secondary AML according to WHO 2016 criteria[2] and have exhausted standard therapeutic options for AML during treatment prior to transformation; or<br>• High-risk or very high-risk MDS according to WHO 2016 criteria[2] and the revised IPSS-R[3] with R/R disease and have exhausted or are ineligible for standard therapeutic options	≥3 RBC units transfused within 16 weeks before C1D1 (pre-transfusion haemoglobin <9.0 g/dL to count towards 3 units total[4])<br>• R/R to erythropoietin stimulation, or endogenous serum erythropoietin level >500 mU/mL [a]

ECOG-PS	Grade 0 or 1	
Haematology laboratory parameters	WBC ≤20 × 10^9/L (hydroxyurea could be used to lower WBC count at screening and during study	
	Platelets ≥10 × 10^9/L	• Absolute neutrophil count ≥0.75 × 10^9/L (ie, ≥750/mm^3) independent of growth factor support for ≥7 days or 7 half-lives, whichever is longer<br>• Platelets ≥50 × 10^9/L (ie, ≥50,000/mm^3) independent of platelet transfusion support for ≥3 days
Chemistry laboratory parameters	• Aspartate aminotransferase and alanine aminotransferase ≤3 × ULN<br>• Serum total bilirubin ≤2 × ULN. Patients with elevated bilirubinemia, such as Gilbert's syndrome, may have been enrolled if conjugated bilirubin was within normal range<br>• Estimated or measured glomerular filtration rate[5] ≥40 mL/min/1.73 m^2	
	Potassium, uric acid, and phosphorus within or below, and calcium within or above, normal range and considered not clinically significant	

Pregnancy	• People of childbearing potential must have had a negative highly sensitive serum (β-human chorionic gonadotropin) at screening and again within 48 hours before the first dose of study treatment<br>• People of childbearing potential must have agreed to the following during the study and for 6 months after the last dose of study treatment: use a barrier method of contraception, use a highly effective preferably user-independent method of contraception, not to donate eggs or freeze for future use for the purposes of assisted reproduction, not plan to become pregnant, and not to breastfeed<br>• During the study and for 90 days after the last dose of study treatment, males must have agreed to: wear a condom when engaging in any activity that allows for passage of ejaculate to another person, not to donate sperm or freeze for future use for the purpose of reproduction, and not plan to father a child
Procedures	• Must have signed an informed consent<br>• Must have been willing and able to adhere to the prohibitions and restrictions specified in the protocol
**Exclusion criteria**	
Acute promyelocytic leukaemia according to WHO 2016 criteria[2]	
Known central nervous system involvement	
Prior solid organ transplantation	
Allogeneic stem cell transplantation<br>• Prior transplantation ≤3 months before first dose of study treatment<br>• Active graft versus host disease from prior transplantation or requirement for immunosuppressant therapy (exception: daily doses ≤10 mg prednisone or equivalent were allowed for adrenal replacement)	
Prior permanent discontinuation of (mono-R/R) or venetoclax (JNJ+VEN) due to toxicity	
Prior treatment with a DHODH inhibitor for an oncology indication or intolerance to a DHODH inhibitor given for non-oncology indication	
Chemotherapy, targeted therapy, immunotherapy, radiotherapy, ESA therapy, luspatercept, or treatment with an investigational drug, within 2 weeks prior to first dose or ≥4 half-lives, whichever is less, or currently receiving investigational therapy in a clinical trial	

Toxicities (except for alopecia, peripheral neuropathy, thrombocytopenia, neutropenia, anaemia) from previous anticancer therapies that did not resolve to baseline or to ≤Grade 1
Known allergies, hypersensitivity, or intolerance to JNJ-74856665, AZA, VEN, or the excipients of these treatments
Cardiovascular disease uncontrolled or diagnosed within the 6 months before first study treatment dose including, but not limited to:<br>• Myocardial infarction<br>• Severe or unstable angina<br>• Clinically significant cardiac arrhythmias<br>• Stroke or transient ischaemic attack<br>• Venous thromboembolic events (eg, pulmonary embolism) within 1 month prior to the first dose of study treatment; uncomplicated (Grade ≤2) deep vein thrombosis is not considered exclusionary<br>• Congestive heart failure (New York Heart Association class III to IV)[6]<br>• Pericarditis or clinically significant pericardial effusion<br>• Myocarditis<br>• Endocarditis
Pulmonary compromise that requires the need for supplemental oxygen use to maintain adequate oxygenation
Administration of a live, attenuated vaccine within 4 weeks before first administration of study treatment or planned within 4 weeks after the last dose of JNJ-74856665. Administration of non-live, attenuated investigational vaccines within 2 weeks before first administration of study treatment. Non-live vaccines granted emergency use authorization and locally approved COVID-19 vaccines were permitted
Temperature >100.4ºF/38ºC at multiple time points within 48 hours before first dose of study treatment
Known to have a positive test result for human immunodeficiency virus unless viral load is undetectable and CD4 count >200 on stable highly active antiretroviral therapy
Active or chronic hepatitis B or hepatitis C infection
Major surgery (eg, requiring general anaesthesia) within 2 weeks before first dose of study treatment or had not recovered from surgery

Any serious underlying medical or psychiatric condition, such as seizure disorder or psychiatric conditions (eg, alcohol or drug abuse), dementia, or altered mental status
Evidence within 7 days before first study treatment dose of active viral, bacterial, or uncontrolled systemic fungal infection requiring parenteral treatment
Required a prohibited medication that could not be discontinued or substituted, or temporally interrupted during the study
Inability to take orally administered drug, or medical disorder or prior surgical resection that may have affected study treatment absorption
Any condition for which, in the opinion of the investigator, participation was not in the best interest of the patient (eg, compromise the well-being) or that could have prevented, limited, or confounded protocol-specified assessments
Uncontrolled (persistent) hypertension: systolic blood pressure >180 mm Hg; diastolic blood pressure >100 mm Hg

AML, acute myeloid leukaemia; AZA, azacitidine; C, cycle; CMML-2, chronic myelomonocytic leukaemia group 2; D, day; DHODH, dihydroorotate dehydrogenase; ECOG-PS, Eastern Cooperative Oncology Group Performance Status; IPPS, International Prognostic Scoring System; JNJ, JNJ-74856665; MDS, myelodysplastic syndrome; RBC, red blood cell; R/R, relapsed or refractory; ULN, upper limit of normal; VEN, venetoclax; WBC, white blood cell; WHO, World Health Organization.

[a] Exception: Del(5q) karyotype was allowed, provided prior treatment with lenalidomide failed or patient was ineligible to receive lenalidomide.

The mono-R/R arm included a cohort to evaluate comedication with a strong CYP3A4 inhibitor, posaconazole. Patients with R/R AML or R/R high- or very-high–risk [3] (HR)-MDS were eligible for all arms except mono-LR, which included patients with R/R very-low, low, or intermediate-risk (LR)-MDS. Patients with R/R CMML-2 were eligible for mono-R/R and JNJ+AZA arms, and patients with newly diagnosed AML or HR-MDS were eligible for the JNJ+AZA arm. The study protocol was approved according to each participating country's process and the Declaration of Helsinki and Good Clinical Practice regulations, and was registered (clinicaltrials.gov, NCT04609826; Eudra-CT, 2020-002375-35). All patients provided written informed consent before study participation.

Therapies disallowed during the study

Any chemotherapy (excluding hydroxyurea), anticancer immunotherapy, experimental therapy (other than JNJ-74856665), or radiotherapy within 2 weeks prior to the first dose or ≥4 half-lives, whichever was less; chronic doses of systemic corticosteroids >10

mg daily of prednisone or equivalent administered for >10 days, unless to manage management of suspected JNJ-74856665-associated AEs; other immunosuppressant agents unless used to treat an AE; and strong inhibitors or inducers of CYP3A4.

Recommended phase 2 dose (RP2D)

A dose-escalation phase, guided by BOIN design (**figure above**) with a target DLT rate of 30%, was intended to determine recommended phase 2 dose(s) (RP2D[s]) and preceded a planned dose-expansion phase (**Figure S1**). The BOIN design was used to help identify recommended phase 2 dose(s) (RP2D[s]). The target DLT rate for the RP2D(s) was 30%. The highest/lowest DLT rates deemed to be underdosing/overdosing were assumed to be 18%/42%. During dose expansion, cumulative data in each RP2D expansion cohort was reviewed at a minimum after every 5th patient completed the DLT-evaluation period. A temporary halt or final stop was triggered if dose de-escalation or elimination thresholds were reached, respectively. Optimal boundaries for dose escalation and de-escalation were 0.236 and 0.359, respectively. Minimum conditions were set for RP2D(s): ≥6 patients were treated at each intended RP2D, and anti-leukaemic activity with ≥1 clinical response (eg, improvement in bone marrow blast count, transfusion independence, or haematologic improvement) was observed.

Safety

Adverse events (AEs) were evaluated using National Cancer Institute Common Terminology Criteria for AE (version 5.0). AEs of special interest (AESI) of any grade included mucositis and diarrhoea. AEs, including DLTs (**table below**), electrocardiograms (ECGs), vital signs, physical examination findings, clinical laboratory test results, and Eastern Cooperative Oncology Group Performance Scale scores informed safety. Peripheral blood and BM samples were collected at baseline and prespecified timepoints for efficacy analyses. Leukaemic blast and stem cells were evaluated centrally using flow cytometry. Response assessments followed European LeukemiaNet 2017 recommendations for AML,[7] with CR with partial haematologic recovery added;[8] Proposed Modified International Working Group Response Criteria for Altering Natural History of MDS;[4,9] and Proposed Modified 2015 International Response Criteria for Myelodysplastic/Myeloproliferative Neoplasms in adults (CMML-2).[10]

Criteria for dose-limiting toxicities

**Criteria for non-haematological toxicity**	**Exceptions**
Grade 3 or 4 laboratory abnormalities	Grade 3 lasting ≤5 days or Grade 4 lasting <24 hours<br>• If not associated with clinical complications<br>• Unless the abnormality is part of a syndrome in which case the grade of the syndrome should dictate the DLT assessment
Grade 3 or 4 tumour lysis syndrome	Recovers in ≤5 days
Concurrent elevations in AST or ALT >3 × ULN, total bilirubin ≥2 × ULN, and alkaline phosphatase ≤2 × ULN with no alternative aetiology (Hy's law)	None
Any other Grade 3 toxicity	Asthenia, anorexia, fever, or constipation lasting <7 days
	Nausea, vomiting or diarrhoea that has recovered in ≤5 days unless requiring tube feeding, total parenteral nutrition, or hospitalization
	Other Grade 3 toxicity that improves to Grade ≤1 or baseline with outpatient treatment in ≤7 days
Any other Grade 4 toxicity	None
Any Grade 5 toxicity	None
**Criteria for haematological toxicity**	**Exceptions**
Grade 4 neutrophil count decreased	Recovers in ≤8 weeks
Any Grade 5 toxicity	None

ALT, alanine aminotransferase; AST, aspartate aminotransferase; DLT, dose-limiting toxicity; ULN, upper limit of normal.

Sampling and pharmacokinetic/pharmacodynamic assessments

Venous blood, plasma, bone marrow (BM), and urine samples were collected at baseline and prespecified timepoints for use in pharmacokinetic/pharmacodynamic analyses. Plasma samples were used to evaluate pharmacokinetics (eg, maximal drug concentration [C_{max}], time-to-peak drug concentration [t_{max}], and area-under-the-concentration [AUC]-time curve) and plasma concentrations of JNJ-74856665, AZA, and VEN. Plasma concentrations were determined using a validated, specific, and sensitive liquid chromatography/mass spectrometry method. JNJ-74856665 pharmacokinetic metrics were compared between monotherapy and combination arms to evaluate drug-drug interactions (DDIs), and DDI with posaconazole comedication on JNJ-74856665 pharmacokinetics was assessed (next section).

Plasma DHO, orotate, and uridine levels were measured as pharmacodynamic markers of target engagement using a liquid chromatography/mass spectrometry-based method following JNJ-74856665 treatment.

Assessment of impact of comedication with posaconazole on the PK of JNJ-74856665

All study requirements and assessments were the same as for other patients except the following. For preliminary food-effect assessment, JNJ-74856665 was administered in the clinic under fasted (reference treatment) or fed (test treatment) condition at therapy initiation (cycle 1 day 1) and cycle 2 day 1, respectively. Mean JNJ-74856665 concentration-time profiles were plotted at steady state in fasting and fed conditions. For assessment of comedication with posaconazole, patients must have been receiving posaconazole for ≥7 days before enrolment to ensure medication plasma level was at steady state, and patients continued preexisting therapy with posaconazole for fungal prophylaxis using standard dosages (200-mg capsules once daily or 300-mg delayed-release tablets) per local practice. Mean JNJ-74856665 concentration-time profiles were plotted at steady state in fasting conditions.

Exploratory clinical study objectives

Exploratory objectives, which were planned but not evaluated because trial enrolment was stopped early, were to explore relationships between pharmacokinetics, pharmacodynamics, adverse event (AE) profile, and preliminary clinical activity of JNJ-74856665 as monotherapy (mono-R/R and mono-LR) and in combination (JNJ+AZA and JNJ+VEN); biomarkers predictive of clinical response of or

resistance to JNJ-74856665 as monotherapy and in combination; nucleotide biosynthesis and salvage pathway metabolites; effect of food on the pharmacokinetics of JNJ-74856665 monotherapy; and effect of comedication with a strong CYP3A4 inhibitor (posaconazole) on the pharmacokinetics of JNJ-74856665 monotherapy.

Statistical analysis

No formal statistical hypothesis testing was conducted. Descriptive statistics (mean [standard deviation (SD)] and median[11]) were used to summarize safety, laboratory, pharmacokinetic, biomarker, and duration-of-response (DOR) findings. AEs were summarized by system organ class, preferred term, worst grade, dose level, and schedule.

Outcomes

Overall response rate (ORR; defined as partial response or better), best overall response (bOR), and number (%) of patients in each disease-response category were tabulated. Since overall responses required a minimum duration of response, additional evaluations of timepoint responses (with no duration requirement) were analyzed separately. Waterfall plots show best change in BM blasts from baseline. Patients were excluded from pharmacokinetic analysis if their data did not allow for accurate assessment. Mean and individual serum JNJ-74856665 concentration-time profiles were plotted after initial study treatment dosing. Biomarker analysis over time was summarized by treatment group.

Sample sizes and calculations

We planned to enroll approximately 281 evaluable patients: approximately 60 in the mono-R/R arm and 20 each in the JNJ+AZA, JNJ+VEN, and mono-LR arms; approximately 46 each in the mono-R/R (A2), JNJ+AZA (B2), and JNJ+VEN (C2) arms; and 23 in the mono-LR (D2) arm. During each RP2D cohort expansion, approximately 23 patients were to be treated to further assess safety and preliminary antitumour activity. A sample size of 20 patients treated at RP2D(s) would provide >95% probability of observing at ≥1 AE with a true incidence rate of ≥15%.

Analysis population definitions

The all-treated population comprised patients who received ≥1 dose of JNJ-74856665 and was used in all safety summaries. The DLT-evaluable population was subset of the all-treated population and included all patients who received any dose of JNJ-74856665 in the DLT evaluation period. Patients must have received ≥75% of the JNJ-74856665 dose in the mono-R/R arm and ≥75% of both study treatments in the JNJ+AZA arm during the DLT-observation period. Patients who had dose interruption for reasons other than study treatment toxicity that resulted in not completing ≥75% of the JNJ-74856665 dose in the mono-R/R and mono-LR arms and ≥75% of both study treatments in the JNJ+AZA and JNJ+VEN arms during the DLT-observation period were to be evaluated and determined to be included based on the length of consecutive treatment doses following the interruption. The pharmacokinetic population comprised all patients who receive ≥1 dose of JNJ-74856665 and have ≥1 evaluable concentration measurement of JNJ-74856665, and the biomarker population consisted of all patients who received ≥1 dose of JNJ-74856665 and had ≥1 pre- or post-treatment biomarker measurement.

Clinical study: Results

Patients

Median patient age was 73 years, and 101 (66.0%) patients were male (**Table 1**). Eighty-nine (58.2%) patients were treated with JNJ-74856665 monotherapy (mono-R/R: 84 [AML, 66 (78.6%); HR-MDS, 18 (21.4%)]; mono-LR: 5). Among 35 (22.9%) JNJ+AZA-treated patients, 20 (57.1%) had AML, 14 (40%) had HR-MDS, and 1 (2.9%) had CMML-2. Among 29 (18.9%) JNJ+VEN-treated patients, 24 (82.8%) had AML and 5 (17.2%) had HR-MDS. Median (range) number of prior lines of therapy across treatment arms was 2 (1.0–7.0). Twenty-five (29.7%) and 10 (28.6%) patients in the mono-R/R and JNJ+VEN arms, respectively, were heavily pretreated (≥3 prior lines), and 9 (25.7%) and 2 (40.0%) patients in the JNJ+AZA and mono-LR arms had no prior AML or LR-MDS therapy, respectively. Both LR-MDS patients were considered refractory due to marked baseline erythropoietin elevations. Analysis populations, disposition, and additional patient demographic and clinical characteristics are shown in **Tables S3, S4, and S5**, respectively.

Table S3: Analysis populations.

	Mono-R/R	JNJ+AZA	JNJ+VEN	Mono-LR	Total
All treated/safety, n (%)	84 (100)	35 (100.0)	29 (100)	5 (100.0)	153 (100.0)
Cohort 1	0.3 mg, *n* = 3	--	0.9 mg + VEN (28 days), *n* = 10	0.9 mg, *n* = 4	--
Cohort 2	0.9 mg, *n* = 14	2.6 mg + AZA, *n* = 6	0.9 mg + VEN (21 days), *n* = 6	2.0 mg, *n* = 1	--
Cohort 3	2.6 mg, *n* = 11	2.6 mg + AZA, *n* = 11	2.0 mg + VEN (21 days), *n* = 11	--	--
Cohort 4	4.0 mg, *n* = 12	4.0 mg + AZA, *n* = 8	4.0 mg + VEN (21 days), *n* = 2	--	--
Cohort 5	6.0 mg, *n* = 2	1.0 mg + AZA, *n* = 10	--	--	--
Cohort 6	5.0 mg, *n* = 19	--	--	--	--
Cohort 7	10.6 mg, *n* = 9	--	--	--	--
Cohort 8	7.0 mg, *n* = 3	--	--	--	--
Cohorts 9 and 10	8.4 mg, *n* = 11	--	--	--	--
Pharmacokinetic, n (%)	84 (100)	32 (91.4)	27 (93.1)	5 (100.0)	148 (96.7)
Biomarker, n (%)	82 (97.6)	35 (100.0)	27 (93.1)	5 (100.0)	149 (97.4)

AML, acute myeloid leukaemia; AZA, azacitidine; CMML-2, chronic myelomonocytic leukaemia group 2; JNJ, JNJ-74856665; LR, lower risk; MDS, myelodysplastic syndrome; R/R, relapsed or refractory; VEN, venetoclax.

65 patients were screen failures.

Mono-R/R and JNJ+VEN: patients with R/R AML who had exhausted or were ineligible for standard therapeutic options, patients with newly transformed secondary AML who exhausted standard therapeutic options during treatment before transformation, and patients with high-risk or very high-risk, R/R MDS who exhausted or were ineligible for standard therapeutic options. The mono-R/R arm also was to include patients with R/R CMML-2 who exhausted or were ineligible for standard therapeutic options.

JNJ+AZA: patients with newly diagnosed or R/R AML who were unsuitable for intensive treatment with a curative intent (including stem cell transplantation) but eligible to receive AZA, patients with high-risk or very high-risk MDS; and patients with CMML-2.

Mono-LR: patients with-very low, low, or intermediate-risk MDS and transfusion dependence.

Table S4: Patient disposition (all-treated/safety populations).

	Mono-R/R	JNJ+AZA	JNJ+VEN	Mono-LR	Total
n	84	35	29	5	153
JNJ-74856665 treatment ongoing, *n* (%)	2 (2.4)	11 (31.4)	9 (31.0)	4 (80.0)	26 (17.0)
Discontinued JNJ-74856665 treatment, *n* (%)	82 (97.6)	24 (68.6)	20 (69.0)	1 (20.0)	127 (83.0)
Reason for discontinuation of JNJ-74856665 treatment [a], *n* (%)					
Adverse event	33 (40.2)	13 (54.2)	7 (35.0)	0 (0.0)	53 (41.7)
Physician decision	18 (22.0)	6 (25.0)	2 (10.0)	0 (0.0)	26 (20.5)
Progressive disease	25 (30.5)	1 (4.2)	7 (35.0)	0 (0.0)	33 (26.0)
Patient refused further study treatment	4 (4.9)	4 (16.7)	2 (10.0)	1 (100.0)	11 (8.7)
Other	2 (2.4)	0 (0.0)	2 (10.0)	0 (0.0)	4 (3.1)
Treatment duration, days [b]					
Mean (SD)	69.6 (72.52)	96.1 (90.17)	64.8 (46.37)	151.4 (97.66)	77.5 (75.25)
Median (range)	50 (3-442)	54 (3-362)	51 (5-170)	147 (23-295)	52 (3-442)
Study ongoing, [c] *n* (%)	9 (10.7)	17 (48.6)	16 (55.2)	5 (100.0)	47 (30.7)
Study completed, *n* (%)	67 (79.8)	14 (40.0)	10 (34.5)	0 (0.0)	91 (59.5)
Discontinued study, *n* (%)	8 (9.5)	4 (11.4)	3 (10.3)	0 (0.0)	15 (9.8)
Reason for discontinuation/completion of study [d], n (%)					
Death	58[e] (77.3)	9 (50.0)	9 (69.2)	-	76 (71.7)
Lost to follow-up	2 (2.7)	1 (5.6)	0 (0.0)	-	3 (2.8)
Withdrawal by patient	6 (8.0)	3 (16.7)	3 (23.1)	-	12 (11.3)
Completed 6 months of follow-up, *n* (%)	9 (12.0)	5 (27.8)	1 (7.7)	-	15 (14.2)
Study duration, days					
Mean (SD)	129.4 (97.65)	146.3 (91.03)	79.5 (47.76)	-	126.1 (93.10)

Median (range)	106 (7-346)	138.5 (24-343)	72 (24-180)	-	99 (7-346)

AML, acute myeloid leukaemia; CMML-2, chronic myelomonocytic leukaemia group 2; JNJ, JNJ-74856665; LR, lower risk; MDS, myelodysplastic syndrome; R/R, relapsed or refractory; SD, standard deviation.

[a] Percentages calculated with the number of discontinued study treatment in each group as denominator.

[b] Total duration of treatment defined as (date of last dose of any study agent–date of first dose of any study agent) + 1.

[c] One patient in the JNJ+AZA arm was erroneously documented as ongoing at the point of data cut-off; retroactive confirmation of death was reported after data cut-off.

[d] Percentages calculated with the number of discontinued/completed study in each group as denominator. One patient discontinued the study to have end-of-life care, which superseded death as a reason for study discontinuation.

Mono-R/R and JNJ+VEN: patients with R/R AML who had exhausted or were ineligible for standard therapeutic options, patients with newly transformed secondary AML who exhausted standard therapeutic options during treatment before transformation, and patients with high-risk or very high-risk, R/R MDS who exhausted or were ineligible for standard therapeutic options. The mono-R/R arm also was to include patients with R/R CMML-2 who exhausted or were ineligible for standard therapeutic options.

JNJ+AZA: patients with newly diagnosed or R/R AML who were unsuitable for intensive treatment with a curative intent (including stem cell transplantation) but eligible to receive AZA, patients with high-risk or very high-risk MDS; and patients with CMML-2.

Mono-LR: patients with very-low, low, or intermediate-risk MDS and transfusion dependence.

Table S5: Patient demographic and clinical characteristics—continued (all-treated/safety population).

	Mono-R/R	JNJ+AZA	JNJ+VEN	Mono-LR	Total
n	84	35	29	5	153
Weight, kg					
Mean (SD)	70.25 (13.303)	73.43 (19.638)	69.54 (12.790)	80.98 (10.985)	71.20 (14.884)
Median (range)	70.7 (41.9-106.3)	70 (40.7-141.7)	68 (42.0-96.0)	80.4 (70.6-97.0)	70.4 (40.7-141.7)
Height, cm					
n	83	34	29	5	151

Mean (SD)	167.78 (10.390)	165.23 (9.774)	168.74 (10.545)	167.00 (9.083)	167.37 (10.222)
Median (range)	169 (137.0-191.0)	164.7 (145.0-183.0)	170 (143.0-193.0)	166 (155.0-179.0)	169 (137.0-193.0)
ECOG-PS, *n* (%)					
0	19 (22.6)	12 (34.3)	5 (17.2)	2 (40.0)	38 (24.8)
1	65 (77.4)	23 (65.7)	24 (82.8)	3 (60.0)	115 (75.2)
Interval from last prior line of therapy, [a] days					
Mean (SD)	87.63 (180.057)	126.27 (173.817)	223.83 (312.815)	166.00 (187.489)	124.18 (217.366)
Median (range)	44 (1.0-1416.0)	80 (1-909)	53 (1-1148)	114 (10-374)	49.5 (1-1416)
Prior HSCT, n (%)					
Yes	12 (14.3)	1 (2.9)	7 (24.1)	0 (0.0)	20 (13.1)
Type of transplant					
Allogeneic HSCT	11 (13.1)	0 (0.0)	6 (20.7)	0 (0.0)	17 (11.1)
Allogeneic HSCT/ autologous HSCT	0 (0.0)	0 (0.0)	1 (3.4)	0 (0.0)	1 (0.7)
Autologous HSCT	0 (0.0)	1 (2.9)	0 (0.0)	0 (0.0)	1 (0.7)
Unknown	1 (1.2)	0 (0.0)	0 (0.0)	0 (0.0)	1 (0.7)
No	72 (85.7)	34 (97.1)	22 (75.9)	5 (100.0)	133 (86.9)
Number of treatment cycles, *n* (%)					
≥1 cycle	84 (100.0)	35 (100.0)	29 (100.0)	5 (100.0)	153 (100.0)
≥2 cycles	61 (72.6)	29 (82.9)	23 (79.3)	5 (100.0)	118 (77.1)
≥3 cycles	49 (58.3)	17 (48.6)	12 (41.4)	4 (80.0)	82 (53.6)
≥4 cycles	29 (34.5)	14 (40.0)	8 (27.6)	4 (80.0)	55 (35.9)
≥5 cycles	23 (27.4)	12 (34.3)	5 (17.2)	4 (80.0)	44 (28.8)
≥6 cycles	17 (20.2)	9 (25.7)	3 (10.3)	4 (80.0)	33 (21.6)

≥7 cycles	12 (14.3)	8 (22.9)	1 (3.4)	3 (60.0)	24 (15.7)
≥8 cycles	10 (11.9)	5 (14.3)	0 (0.0)	2 (40.0)	17 (11.1)
Mean (SD)	3.8 (3.48)	4.0 (3.19)	2.8 (1.68)	7.6 (4.72)	3.8 (3.27)
Median (range)	3 (1-22)	2 (1-13)	2 (1-7)	7 (2-15)	3 (1-22)
Total dose received, mg					
Mean (SD)	152.6 (144.46)	159.9 (207.24)	52.9 (32.65)	134.8 (84.40)	134.8 (151.62)
Median (range)	113 (6-628)	67.6 (3-732)	52 (2-104)	132.3 (44-266)	81 (2-732)
Total number of administrations					
Mean (SD)	46.6 (39.57)	62.3 (58.94)	40.5 (29.58)	144.4 (101.47)	52.2 (49.10)
Median (range)	39 (3-177)	40 (3-241)	33 (2-109)	147 (22-295)	37 (2-295)
Dose intensity (mg/day) [b]					
Mean (SD)	2.66 (1.49)	1.59 (0.91)	0.90 (0.58)	1.05 (0.49)	2.03 (1.42)
Median (range)	2.60 (0.2-7.3)	1.68 (0.4-4.0)	0.62 (0.3-2.7)	0.90 (0.7-1.9)	1.78 (0.2-7.3)

AZA, azacitidine; AML, acute myeloid leukaemia; CMML-2, chronic myelomonocytic leukaemia group 2; ECOG-PS, Eastern Cooperative Oncology Group Performance Status; HSCT, haematopoietic stem cell transplant; JNJ, JNJ-74856665; LR, lower risk; MDS, myelodysplastic syndrome; R/R, relapsed or refractory; SD, standard deviation; VEN, venetoclax.

[a] Interval from last prior line of therapy was defined as the time between treatment start date and date of last prior line of therapy.

[b] Dose intensity was defined as the total dose received divided by the duration of treatment (days).

Percentages were calculated based on the number of patients in each group as denominator. A patient was considered as treated in a cycle if they received any nonzero dose of JNJ-74856665 in that cycle.

Mono-R/R and JNJ+VEN: patients with R/R AML who had exhausted or were ineligible for standard therapeutic options, patients with newly transformed secondary AML who exhausted standard therapeutic options during treatment before transformation, and patients with high-risk or very high-risk, R/R MDS who exhausted or were ineligible for standard therapeutic options. The mono-R/R arm also was to include patients with R/R CMML-2 who exhausted or were ineligible for standard therapeutic options.

JNJ+AZA: patients with newly diagnosed or R/R AML who were unsuitable for intensive treatment with a curative intent (including stem cell transplantation) but eligible to receive AZA, patients with high-risk or very high-risk MDS-and patients with CMML-2.
Mono-LR: patients with very-low, low, or intermediate-risk MDS and transfusion dependence.

MTD and RP2D of JNJ-74856665

JNJ-74856665 maximum administered doses were 10.0 mg for mono-R/R, 2.0 mg for mono-LR, and 4.0 mg for JNJ+AZA and JNJ+VEN (**Figure S6**).

Figure S6: Dosing escalation schema (all-treated population).

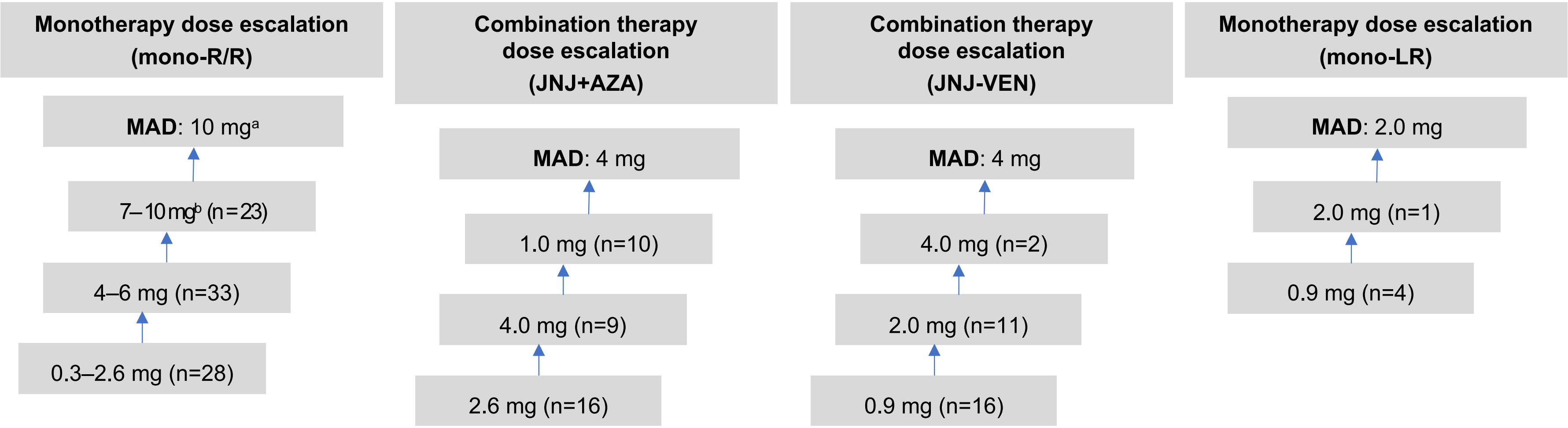

AML, acute myeloid leukaemia; AZA, azacitidine; BOIN, Bayesian optimal interval; CMML-2, chronic myelomonocytic leukaemia group 2; JNJ, JNJ-74856665; LR, lower risk; MAD, maximal administered dose; MDS, myelodysplastic syndrome; VEN, venetoclax.
Mono-R/R and JNJ+VEN: patients with R/R AML who had exhausted or were ineligible for standard therapeutic options, patients with newly transformed secondary AML who exhausted standard therapeutic options during treatment before transformation, and patients with high-risk or very high-risk, R/R MDS who exhausted or were ineligible for standard therapeutic options. Mono-R/R also was to include patients with R/R CMML-2 who exhausted or were ineligible for standard therapeutic options.

JNJ+AZA: patients with newly diagnosed or R/R AML who were unsuitable for intensive treatment with a curative intent (including stem cell transplantation) but eligible to receive AZA, patients with high-risk or very high-risk MDS; and patients with CMML-2.
Mono-LR: patients with very-low, low, or intermediate-risk MDS and transfusion dependence.

Safety

In the mono-R/R arm, most common Grade ≥3 TEAE was anaemia (26 [31%]), and most common JNJ-74856665-related Grade ≥3 TEAEs were stomatitis (17 [20.2%]) and thrombocytopenia (11 [13.1%]) (**Table S6**). One patient in the mono-LR arm experienced Grade ≥3 TEAEs (mouth ulceration, norovirus gastroenteritis, *Klebsiellas pp* infection, and hidradenitis) not considered related to JNJ-74856665. With JNJ+AZA, most common Grade ≥3 TEAEs were thrombocytopenia (15 [42.9%]: 5 [14.3%] JNJ-74856665 related [**Table S6**]) and anaemia (11 [31.4%]; 3 [8.6%] JNJ-74856665 related [**Table S6**]). Most common Grade ≥3 TEAEs with JNJ+VEN were neutropenia (8 [27.6%]), anaemia (7 [24.1%]), and thrombocytopenia (6 [20.7%]), and most common JNJ-74856665-related Grade ≥3 TEAEs were stomatitis (3 [10.3%]) and thrombocytopenia (3 [10.3%]) (**Table S6**). Stomatitis was the most common JNJ-74856665-related SAE (mono-R/R, 12 [14.3%]; JNJ+AZA, 3 [10.3%]) (**Table S7**). Maculopapular rash was observed as an SAE only in JNJ+AZA recipients (2 [5.7%]).

Table S6: Patients with treatment-emergent adverse events and with Grade ≥3 adverse events considered related to JNJ-74856665 by investigators (safety population).

	Mono-R/R	JNJ+AZA	JNJ+VEN	Mono-LR
n	84	35	29	5
Patients with ≥1 TEAE, n (%)				
AE	81 (96.4)	35 (100.0)	28 (96.6)	5 (100.0)
Related AEs [a, b]	60 (71.4)	30 (85.7)	16 (55.2)	3 (60.0)
Related to JNJ-74856665 AEs [a]	60 (71.4)	23 (65.7)	13 (44.8)	3 (60.0)
Related non-serious AEs [a, b]	60 (71.4)	29 (82.9)	15 (51.7)	3 (60.0)

Related to JNJ-74856665 non-serious AEs [a]	60 (71.4)	23 (65.7)	12 (41.4)	3 (60.0)
AE leading to death [c]	29 (34.5)	4 (11.4)	8 (27.6)	0 (0.0)
Related AEs leading to death [a, b, c]	1 (1.2)	1 (2.9)	0 (0.0)	0 (0.0)
Related to JNJ-74856665 AEs leading to death [a, c]	1 (1.2)	1 (2.9)	0 (0.0)	0 (0.0)
Serious AEs	61 (72.6)	25 (71.4)	22 (75.9)	1 (20.0)
Related serious AEs [a, b]	16 (19.0)	9 (25.7)	5 (17.2)	0 (0.0)
Related to JNJ-74856665 serious AEs [a]	16 (19.0)	6 (17.1)	4 (13.8)	0 (0.0)
Maximum toxicity of any TEAE				
Grade 1	2 (2.4)	0 (0)	1 (3.4)	2 (40.0)
Grade 2	10 (11.9)	2 (5.7)	2 (6.9)	2 (40.0)
Grade 3	19 (22.6)	15 (42.9)	11 (37.9)	1 (20.0)
Grade 4	21 (25.0)	14 (40.0)	6 (20.7)	0 (0.0)
Grade 5	29 (34.5)	4 (11.4)	8 (27.6)	0 (0.0)
AE leading to discontinuation	36 (42.9)	12 (34.3)	7 (24.1)	0 (0.0)
Related AEs leading to discontinuation [a,b]	12 (14.3)	9 (25.7)	1 (3.4)	0 (0.0)
Related to JNJ-74856665 AEs leading to discontinuation [a]	12 (14.3)	5 (14.3)	1 (3.4)	0 (0.0)
Any dose-limiting toxicity TEAE	12 (14.3)	4 (11.4)	4 (13.8)	0 (0.0)
Maximum toxicity of drug-related TEAE [a, b]				
Grade 1 or 2	31 (36.9)	12 (34.3)	5 (17.2)	3 (60.0)
≥Grade 3	29 (34.5)	18 (51.4)	11 (37.9)	0 (0.0)
Maximum toxicity of JNJ-74856665-related TEAE [a]				
Grade 1 or 2	31 (36.9)	12 (34.3)	5 (17.2)	3 (60.0)
≥Grade 3	29 (34.5)	11 (31.4)	8 (27.6)	0 (0.0)
Patients with ≥1 TE toxicity Grade ≥3 AE related to JNJ-7485665, [a] n (%)	29 (34.5)	11 (31.4)	8 (27.6)	0 (0.0)

Gastrointestinal disorders	18 (21.4)	2 (5.7)	4 (13.8)	--
Stomatitis	17 (20.2)	--	3 (10.3)	--
Anal inflammation	2 (2.4)	--	--	--
Diarrhoea	1 (1.2)	1 (2.9)	1 (3.4)	--
Neutropenic colitis	--	1 (2.9)	--	--
Proctalgia	--	1 (2.9)	--	--
Blood and lymphatic system disorders	12 (14.3)	7 (20.0)	3 (10.3)	--
Thrombocytopenia	11 (13.1)	5 (14.3)	3 (10.3)	--
Anaemia	6 (7.1)	3 (8.6)	2 (6.9)	--
Neutropenia	2 (2.4)	1 (2.9)	2 (6.9)	--
Febrile neutropenia	--	1 (2.9)	--	--
General disorders and administration-site conditions	2 (2.4)	2 (5.7)	--	--
Asthenia	2 (2.4)	--	--	--
Fatigue	--	--	1 (3.4)	--
Infections and infestations	--	2 (5.7)	--	--
Bronchopulmonary aspergillosis	--	1 (2.9)	--	--
Pneumonia	--	1 (2.9)	--	--
Septic shock	--	1 (2.9)	--	--
Investigations	1 (1.2)	--	--	--
Lipase increased	1 (1.2)	--	--	--
Neoplasms benign, malignant and unspecified (including cysts and polyps)	1 (1.2)	--	--	--
Differentiation syndrome	1 (1.2)	--	--	--
Reproductive system and breast disorders	1 (1.2)	--	--	--
Vulvovaginal inflammation	1 (1.2)	--	--	--

Respiratory, thoracic, and mediastinal disorders	1 (1.2)	1 (2.9)	--	--
Aspiration	1 (1.2)	--	--	--
Pulmonary haemorrhage	--	1 (2.9)	--	--
Skin and subcutaneous tissue disorders	1 (1.2)	2 (5.7)	--	--
Stevens-Johnson syndrome	1 (1.2)	--	--	--
Rash maculo-papular	--	2 (5.7)	--	--

AE, adverse event; AML, acute myeloid leukaemia; CMML-2, chronic myelomonocytic leukaemia group 2; JNJ, JNJ-74856665; LR, lower risk; MDS, myelodysplastic syndrome; R/R, relapsed or refractory; TE, treatment-emergent; VEN, venetoclax.

[a] An AE was assessed by the investigator as related to study agent.

[b] AEs related to ≥1 of the 3 study drugs.

Patients were counted only once for any given event, regardless of the number of times they actually experienced the event.

AEs were reported using Medical Dictionary for Regulatory Activities version 25.1.

Table S7: Treatment-emergent serious adverse events occurring in ≥2 patients (safety population).

	**Mono-R/R**	**JNJ+AZA**	**JNJ+VEN**	**Mono-LR**	**Total**
n	84	35	29	5	153
Patients with ≥1 event, *n* (%)	61 (72.6)	25 (71.4)	22 (75.9)	1 (20.0)	109 (71.2)
Pneumonia	10 (11.9)	3 (8.6)	3 (10.3)	0 (0.0)	16 (10.5)
Stomatitis	12 (14.3)	0 (0.0)	3 (10.3)	0 (0.0)	15 (9.8)
Febrile neutropenia	7 (8.3)	2 (5.7)	3 (10.3)	0 (0.0)	12 (7.8)
General physical health deterioration	9 (10.7)	0 (0.0)	3 (10.3)	0 (0.0)	12 (7.8)
COVID-19	4 (4.8)	1 (2.9)	2 (6.9)	0 (0.0)	7 (4.6)
Pyrexia	3 (3.6)	2 (5.7)	2 (6.9)	0 (0.0)	7 (4.6)
Septic shock	4 (4.8)	1 (2.9)	0 (0.0)	0 (0.0)	5 (3.3)

Bronchopulmonary aspergillosis	0 (0.0)	4 (11.4)	0 (0.0)	0 (0.0)	4 (2.6)
Cellulitis	3 (3.6)	0 (0.0)	1 (3.4)	0 (0.0)	4 (2.6)
Anaemia	2 (2.4)	1 (2.9)	0 (0.0)	0 (0.0)	3 (2.0)
Escherichia sepsis	3 (3.6)	0 (0.0)	0 (0.0)	0 (0.0)	3 (2.0)
Klebsiella sepsis	2 (2.4)	1 (2.9)	0 (0.0)	0 (0.0)	3 (2.0)
Lower respiratory tract infection	0 (0.0)	2 (5.7)	1 (3.4)	0 (0.0)	3 (2.0)
Multiple organ dysfunction syndrome	2 (2.4)	0 (0.0)	1 (3.4)	0 (0.0)	3 (2.0)
Pneumonia fungal	1 (1.2)	0 (0.0)	2 (6.9)	0 (0.0)	3 (2.0)
Prostatitis Escherichia coli	1 (1.2)	1 (2.9)	1 (3.4)	0 (0.0)	3 (2.0)
Respiratory arrest	2 (2.4)	1 (2.9)	0 (0.0)	0 (0.0)	3 (2.0)
Sepsis	2 (2.4)	0 (0.0)	1 (3.4)	0 (0.0)	3 (2.0)
Staphylococcal sepsis	3 (3.6)	0 (0.0)	0 (0.0)	0 (0.0)	3 (2.0)
Thrombocytopenia	1 (1.2)	1 (2.9)	1 (3.4)	0 (0.0)	3 (2.0)
Cardiac failure	2 (2.4)	0 (0.0)	0 (0.0)	0 (0.0)	2 (1.3)
Diarrhoea	1 (1.2)	1 (2.9)	0 (0.0)	0 (0.0)	2 (1.3)
Escherichia bacteremia	0 (0.0)	2 (5.7)	0 (0.0)	0 (0.0)	2 (1.3)
Escherichia urinary tract infection	2 (2.4)	0 (0.0)	0 (0.0)	0 (0.0)	2 (1.3)
Infection	0 (0.0)	1 (2.9)	1 (3.4)	0 (0.0)	2 (1.3)
Leucocytosis	2 (2.4)	0 (0.0)	0 (0.0)	0 (0.0)	2 (1.3)
Neutropenic colitis	0 (0.0)	2 (5.7)	0 (0.0)	0 (0.0)	2 (1.3)
Pericarditis	2 (2.4)	0 (0.0)	0 (0.0)	0 (0.0)	2 (1.3)
Rash maculopapular	0 (0.0)	2 (5.7)	0 (0.0)	0 (0.0)	2 (1.3)
Respiratory tract infection fungal	1 (1.2)	0 (0.0)	1 (3.4)	0 (0.0)	2 (1.3)
Tooth infection	1 (1.2)	1 (2.9)	0 (0.0)	0 (0.0)	2 (1.3)

Urosepsis	0 (0.0)	1 (2.9)	1 (3.4)	0 (0.0)	2 (1.3)
Patients with ≥1 TEAE related to JNJ-74856665, *n* (%)	16 (19.0)	6 (17.1)	4 (13.8)	0 (0.0)	26 (17.0)
Stomatitis	12 (14.3)	0 (0.0)	3 (10.3)	0 (0.0)	15 (9.8)
Diarrhoea	1 (1.2)	1 (2.9)	0 (0.0)	0 (0.0)	2 (1.3)
Rash maculopapular	0 (0.0)	2 (5.7)	0 (0.0)	0 (0.0)	2 (1.3)

AE, adverse event; AML, acute myeloid leukaemia; AZA, azacitidine; CMML-2, chronic myelomonocytic leukaemia group 2; JNJ, JNJ-74856665; LR, lower risk; MDS, myelodysplastic syndrome; R/R, relapsed or refractory; TEAE, treatment-emergent AE; VEN, venetoclax.

Patients were counted only once for any given event.

AEs were reported using Medical Dictionary for Regulatory Activities version 25.1.

Serious AEs reported in only 1 patient were abdominal pain, acinetobacter infection, acute kidney injury, anal fistula, anal inflammation, infection, arthritis bacterial, aspergillus infection, aspiration, blood creatinine increased, campylobacter sepsis, catheter site haemorrhage, cellulitis orbital, cerebral haemorrhage, chronic obstructive pulmonary disease, citrobacter infection, COVID-19 pneumonia, dermo-hypodermitis, device related infection, differentiation syndrome, diverticulitis, dyspnea, fatigue, fungal sepsis, gastric haemorrhage, groin infection, heart rate irregular, hepatic function abnormal, hidradenitis, hyponatremia, infective exacerbation of chronic obstructive airways disease, interstitial lung disease, intestinal perforation, klebsiella infection, meningioma, myocardial infarction, myositis, nephritis, neutropenia, oral herpes, otitis externa, otitis media staphylococcal, peritonsillar abscess, pneumocystis, jirovecii pneumonia, pneumonia bacterial, pneumonia klebsiella, pneumonia mycoplasma, presyncope, proctalgia, prostate cancer, pseudomonal sepsis, pulmonary haemorrhage, respiratory syncytial virus infection, skin infection, soft tissue infection, splenic haematoma, splenic infarction, splenomegaly, staphylococcal infection, stenotrophomonas sepsis, Stevens-Johnson syndrome, transfusion with incompatible blood, tumour lysis syndrome, ulna fracture, upper respiratory tract infection, vaginal haemorrhage, vulvovaginal inflammation, wrist fracture.

Serious AEs related to JNJ-74856665 reported in only 1 patient were anaemia, anal inflammation, aspiration, bronchopulmonary aspergillosis, cellulitis, differentiation syndrome, Escherichia bacteremia, fatigue, febrile neutropenia, neutropenic colitis, pulmonary haemorrhage, septic shock, splenic haematoma, Stevens-Johnson syndrome, vulvovaginal inflammation.

Mono-R/R and JNJ+VEN: patients with R/R AML who had exhausted or were ineligible for standard therapeutic options, patients with newly transformed secondary AML who exhausted standard therapeutic options during treatment before transformation, and patients with high-risk or very

high-risk, R/R MDS who exhausted or were ineligible for standard therapeutic options. The mono-R/R arm also was to include patients with R/R CMML-2 who exhausted or were ineligible for standard therapeutic options.

JNJ+AZA: patients with newly diagnosed or R/R AML who were unsuitable for intensive treatment with a curative intent (including stem cell transplantation) but eligible to receive AZA, patients with high-risk or very high-risk MDS; and patients with CMML-2.

Mono-LR: patients with very-low, low, or intermediate-risk MDS and transfusion dependence.

Table S8: Dose-limiting toxicity treatment-emergent adverse events (safety population).

	**Mono-R/R**	**JNJ+AZA**	**JNJ+VEN**	**Mono-LR**
n (%)	84	35	29	5
Patients with ≥1 dose-limiting toxicity treatment-emergent adverse events	12 (14.3)	4 (11.4)	4 (13.8)	0 (0.0)
Gastrointestinal disorders	11 (13.1)	1 (2.9)	3 (10.3)	0 (0.0)
Stomatitis	11 (13.1)	0 (0)	3 (10.3)	0 (0.0)
Anal inflammation	1 (1.2)	0 (0)	0 (0.0)	0 (0.0)
Neutropenic colitis	0 (0.0)	1 (2.9)	0 (0.0)	0 (0.0)
Neoplasms benign, malignant and unspecified, including cysts and polyps	1 (1.2)	0 (0.0)	0 (0.0)	0 (0.0)
Differentiation syndrome	1 (1.2)	0 (0.0)	0 (0.0)	0 (0.0)
Reproductive system and breast disorders	1 (1.2)	0 (0.0)	0 (0.0)	0 (0.0)
Vulvovaginal inflammation	1 (1.2)	0 (0.0)	0 (0.0)	0 (0.0)
Skin and subcutaneous tissue disorders	1 (1.2)	2 (5.7)	0 (0.0)	0 (0.0)
Stevens-Johnson syndrome	1 (1.2)	0 (0.0)	0 (0.0)	0 (0.0)
Rash maculopapular	0 (0.0)	2 (5.7)	0 (0.0)	0 (0.0)
Infections and infestations	0 (0.0)	1 (2.9)	0 (0.0)	0 (0.0)

Septic shock	0 (0.0)	1 (2.9)	0 (0.0)	0 (0.0)
Respiratory, thoracic and mediastinal disorders	0 (0.0)	1 (2.9)	0 (0.0)	0 (0.0)
Pulmonary haemorrhage	0 (0.0)	1 (2.9)	0 (0.0)	0 (0.0)
Blood and lymphatic system disorders	0 (0.0)	0 (0.0)	1 (3.4)	0 (0.0)
Neutropenia	0 (0.0)	0 (0.0)	1 (3.4)	0 (0.0)

AML, acute myeloid leukaemia; AZA, azacitidine; CMML-2, chronic myelomonocytic leukaemia group 2; JNJ, JNJ-74856665; LR, lower risk; MDS, myelodysplastic syndrome; R/R, relapsed or refractory; VEN, venetoclax.

Mono-R/R and JNJ+VEN: patients with R/R AML who had exhausted or were ineligible for standard therapeutic options, patients with newly transformed secondary AML who exhausted standard therapeutic options during treatment before transformation, and patients with high-risk or very high-risk, R/R MDS who exhausted or were ineligible for standard therapeutic options. The mono-R/R arm also was to include patients with R/R CMML-2 who exhausted or were ineligible for standard therapeutic options.

JNJ+AZA: patients with newly diagnosed or R/R AML who were unsuitable for intensive treatment with a curative intent (including stem cell transplantation) but eligible to receive AZA, patients with high-risk or very high-risk MDS; and patients with CMML-2.

Mono-LR: patients with very-low, low, or intermediate-risk MDS and transfusion dependence.

Table S9: Adverse events of special interest (mucositis and diarrhoea) treatment-emergent adverse events (safety population).

	**Mono-R/R**	**JNJ+AZA**	**JNJ+VEN**	**Mono-LR**	**Total**
n	84	35	29	5	153
Patients with ≥1 TEAE of mucositis, [a] *n* (%)	41 (48.8)	7 (20.0)	4 (13.8)	0 (0.0)	52 (34.0)
Maximum toxicity of mucositis, *n* (%)					
Grade 1	6 (7.1)	2 (5.7)	0 (0.0)	0 (0.0)	8 (5.2)
Grade 2	18 (21.4)	5 (14.3)	1 (3.4)	0 (0.0)	24 (15.7)
Grade 3	14 (16.7)	0 (0.0)	3 (10.3)	0 (0.0)	17 (11.1)
Grade 4	3 (3.6)	0 (0.0)	0 (0.0)	0 (0.0)	3 (2.0)
Grade 5	0 (0.0)	0 (0.0)	0 (0.0)	0 (0.0)	0 (0.0)

Mucositis DLTs	11 (13.1)	0 (0.0)	3 (10.3)	0 (0.0)	14 (9.2)
Study day of onset, median (range)	24 (1-358)	14.5 (1-220)	21 (8-29)	-	23 (1-358)
Patients with ≥1 JNJ-74856665-related TEAE of mucositis, [a] *n* (%)	41 (48.8)	6 (17.1)	4 (13.8)	0 (0.0)	51 (33.3)
Maximum toxicity of mucositis, *n* (%)					
Grade 1	6 (7.1)	2 (5.7)	0 (0.0)	0 (0.0)	8 (5.2)
Grade 2	18 (21.4)	4 (11.4)	1 (3.4)	0 (0.0)	23 (15.0)
Grade 3	14 (16.7)	0 (0.0)	3 (10.3)	0 (0.0)	17 (11.1)
Grade 4	3 (3.6)	0 (0.0)	0 (0.0)	0 (0.0)	3 (2.0)
Grade 5	0 (0.0)	0 (0.0)	0 (0.0)	0 (0.0)	0 (0.0)
JNJ-74856665-related mucositis DLTs, *n* (%)	11 (13.1)	0 (0.0)	3 (10.3)	0 (0.0)	14 (9.2)
Study day of onset, median (range)	23.5 (1-358)	15 (1-220)	21 (8-29)	-	23 (1-358)
Patients with ≥1 TEAE of diarrhoea, *n* (%)	27 (32.1)	13 (37.1)	9 (31.0)	1 (20.0)	50 (32.7)
Maximum toxicity of diarrhoea, *n* (%)					
Grade 1	17 (20.2)	9 (25.7)	4 (13.8)	1 (20.0)	31 (20.3)
Grade 2	8 (9.5)	3 (8.6)	4 (13.8)	0	15 (9.8)
Grade 3	2 (2.4)	1 (2.9)	1 (3.4)	0	4 (2.6)
Grade 4	0 (0.0)	0 (0.0)	0 (0.0)	0 (0.0)	0 (0.0)
Grade 5	0 (0.0)	0 (0.0)	0 (0.0)	0 (0.0)	0 (0.0)
Diarrhoea DLTs	0 (0.0)	0 (0.0)	0 (0.0)	0 (0.0)	0 (0.0)
Study day of onset, median (range)	34 (1-132)	31 (1-79)	33 (2-170)	62 (62)	34 (1-170)
Patients with ≥1 JNJ-74856665-related TEAE of diarrhoea, *n* (%)	15 (17.9)	8 (22.9)	3 (10.3)	1 (20.0)	27 (17.6)
Maximum toxicity of diarrhoea, *n* (%)					

Grade 1	9 (10.7)	5 (14.3)	1 (3.4)	1 (20.0)	16 (10.5)
Grade 2	5 (6.0)	2 (5.7)	1 (3.4)	0 (0.0)	8 (5.2)
Grade 3	1 (1.2)	1 (2.9)	1 (3.4)	0 (0.0)	3 (2.0)
Grade 4	0 (0.0)	0 (0.0)	0 (0.0)	0 (0.0)	0 (0.0)
Grade 5	0 (0.0)	0 (0.0)	0 (0.0)	0 (0.0)	0 (0.0)
JNJ-74856665-related diarrhoea DLTs, *n* (%)	0 (0.0)	0 (0.0)	0 (0.0)	0 (0.0)	0 (0.0)
Study day of onset, median (range)	37 (2-132)	30 (1-48)	34 (22-53)	62 (62-62)	35.5 (1-132)

AE, adverse event; AML, acute myeloid leukaemia; AZA*,* azacitidine; CMML-2, chronic myelomonocytic leukaemia group 2; JNJ, JNJ-74856665; LR, lower risk; MDS, myelodysplastic syndrome; R/R, relapsed or refractory; TEAE, treatment-emergent AE; VEN, venetoclax.

[a] Preferred terms included 'anal inflammation,' 'glossitis,' 'mucosal inflammation,' 'pharyngeal inflammation,' 'stomatitis,' and 'vulvovaginal inflammation.'

The worst severity event experienced by the patient was used. If a patient had all AEs with missing severities; the patient was only counted as missing.

A patient could have had both JNJ-74856665-related and JNJ-74856665-unrelated TEAE(s) and, therefore, be included in both the ≥1TEAEs and ≥1JNJ-74856665-related TEAEs calculations.

Study day was relative to the first day of treatment (day 1).

AEs were coded using Medical Dictionary for Regulatory Activities version 25.1.

Mono-R/R and JNJ+VEN: patients with R/R AML who had exhausted or were ineligible for standard therapeutic options, patients with newly transformed secondary AML who exhausted standard therapeutic options during treatment before transformation, and patients with high-risk or very high-risk, R/R MDS who exhausted or were ineligible for standard therapeutic options. The mono-R/R arm also was to include patients with R/R CMML-2 who exhausted or were ineligible for standard therapeutic options.

JNJ+AZA: patients with newly diagnosed or R/R AML who were unsuitable for intensive treatment with a curative intent (including stem cell transplantation) but eligible to receive AZA, patients with high-risk or very high-risk MDS; and patients with CMML-2.

Mono-LR: patients with very-low, low, or intermediate-risk MDS and transfusion dependence.

Continuous daily dosing of ≥6-mg JNJ-74856665 resulted in a $C_{avg, ss}$ of >30 ng/ml, with a >30% probability of Grade ≥2 mucositis.

Table S10: Patients with Grade ≥2 mucositis treatment-emergent adverse events by grade (safety population).

	Mono-R/R				JNJ+AZA		JNJ+VEN		Total			
JNJ-74856665, mg	<2.6	2.6 - 5	>5	Total	2.6 - 5	Total	<2.6	Total	<2.6	2.6 - 5	>5	Total
n	17	42	25	84	25	35	27	29	59	69	25	153
≥1 ≥Grade 2 TEAEs of mucositis, n (%)	4 (23.5)	16 (38.1)	15 (60.0)	35 (41.7)	5 (20.0)	5 (14.3)	4 (14.8)	4 (13.8)	8 (13.6)	21 (30.4)	15 (60.0)	44 (28.8)
Grade 2	3 (17.6)	7 (16.7)	8 (32.0)	18 (21.4)	5 (20.0)	5 (14.3)	1 (3.7)	1 (3.4)	4 (6.8)	12 (17.4)	8 (32.0)	24 (15.7)
Grade 3	1 (5.9)	8 (19.0)	5 (20.0)	14 (16.7)	0	0	3 (11.1)	3 (10.3)	4 (6.8)	8 (11.6)	5 (20.0)	17 (11.1)
Grade 4	0	1 (2.4)	2 (8.0)	3 (3.6)	0	0	0	0	0	1 (1 .4)	2 (8.0)	3 (2.0)
Grade 5	0	0	0	0	0	0		0	0	0	0	0
≥Grade 2 mucositis DLTs, *n* (%)	1 (5.9)	5 (11.9)	5 (20.0)	11 (13.1)	0	0	3 (11.1)	3 (10.3)	4 (6.8)	5 (7.2)	5 (20.0)	14 (9.2)
Study day of onset												
Median	173	34	11	23	12	12	21	21	79	22	11	22
Range	(4; 358)	(4; 157)	(1; 95)	(1; 358)	(1; 15)	(1; 15)	(8; 29)	(8; 29)	(4; 358)	(1; 157)	(1; 95)	(1; 358)

AE, adverse event; AML, acute myeloid leukaemia; AZA*,* azacitidine; CMML-2, chronic myelomonocytic leukaemia group 2; JNJ, JNJ-74856665; LR, lower risk; MDS, myelodysplastic syndrome; R/R, relapsed or refractory; TEAE, treatment-emergent AE; VEN, venetoclax.

No patient in the JNJ-AZA arm who received a JNJ-74856665 dose level of <2.6 mg (*n* = 10) had events, and no patient received a JNJ-74856665 dose level of >5 mg; no patient in the JNJ-VEN arm who received a JNJ-74856665 dose level of 2.6–5 mg (*n* = 10) had events, and no patient received a JNJ-74856665 dose level of >5 mg; and no patient in the mono-LR arm who received a JNJ-74856665 dose level of <2.6 mg (*n* = 5) had events, and no patient received a JNJ-74856665 dose level of 2.6–5 mg or >5 mg.

AEs were reported using Medical Dictionary for Regulatory Activities version 25.1.

Mono-R/R and JNJ+VEN: patients with relapsed or refractory AML who had exhausted or were ineligible for standard therapeutic options, patients with newly transformed secondary AML who exhausted standard therapeutic options during treatment before transformation, and patients with high-

risk or very high-risk, relapsed or refractory MDS who exhausted or were ineligible for standard therapeutic options. The mono-R/R arm also was to include patients with relapsed or refractory CMML-2 who exhausted or were ineligible for standard therapeutic options.
JNJ+AZA: patients with newly diagnosed or R/R disease AML who were unsuitable for intensive treatment with a curative intent (including stem cell transplantation) but eligible to receive AZA, patients with high-risk or very high-risk MDS; and patients with high CMML-2.
Mono-LR: patients with very low, low, or intermediate-risk MDS and transfusion dependence.

In the mono-R/R arm, 36 (42.9%) patients experienced TEAEs leading to discontinuation, including stomatitis (6 [7.1%]); 12 (14.3%)—including all TEAEs of stomatitis—were considered JNJ-74856665 related. Forty-three (51.2%) patients had dose interruption due to TEAEs (eg, stomatitis, 24 [28.6%]; diarrhoea, 5 [6%]), and 20 (23.8%) patients had dose reduction due to TEAEs (eg, stomatitis, 15 [17.9%]). Twelve (34.3%) JNJ+AZA-treated patients discontinued JNJ-74856665 due to TEAEs (eg, bronchopulmonary *Aspergillosis* pneumonia, 2 [5.7%]; maculopapular rash, 2 [5.7%]), with 5 (14.3%) considered JNJ-74856665 related. Twenty-three (65.7%) patients had dose interruption due to ≥1 TEAE, including stomatitis (2 [5.7%]), and 4 (11.4%) patients had dose reduction due to ≥1 TEAE, including stomatitis (3 [8.6%]). Seven (24.1%) JNJ+VEN-treated patients discontinued JNJ-74856665 due to TEAEs. In 1 (3.4%) patient, these TEAEs—mostly gastrointestinal disorders—were considered JNJ-74856665 related. Fifteen (51.7%) and 2 (6.9%) patients had TEAEs leading to dose interruption and reduction, respectively (eg, stomatitis (2 [6.9%] each). Interrupting treatment at toxicity onset helped mitigate severity and shortened duration of recovery, but study therapy ultimately was considered not tolerable because frequent dose adjustments were required.

Seventy-seven (50.3%) patients died during the study—59 (70.2%), 9 (25.7%), and 9 (31.0%) in the mono-R/R, JNJ+AZA, and JNJ+VEN arms, respectively—primarily because of unrelated AEs and PD (**table below**). One mono-R/R patient had a JNJ-74856665-related TEAE of bronchial aspiration (complication of stomatitis) leading to death, and 1 JNJ+AZA-treated patient had a JNJ-74856665-/AZA-related DLT of pulmonary haemorrhage leading to death.

No clinically meaningful changes from baseline were observed in vital signs, physical examination, or clinical laboratory findings, except for clinically significant abnormal ECG findings in 4 patients. In the mono-R/R arm, a Grade 1 QT prolongation occurred that was considered JNJ-74856665 related and spontaneously resolved during the study, and a clinically significant ECG abnormality attributed

to *Escherichia coli* septic shock occurred that was not considered JNJ-74856665 related and did not resolve during the study. One patient with a medical history of bigeminy treated with JNJ+AZA had a clinically significant ECG abnormality that was considered not JNJ-74856665 related and was well-controlled with a pacemaker, and one patient treated with JNJ+VEN had Grade 2 tachycardia that was considered not JNJ-74856665 related and resolved during the study.

Deaths (safety population).

	Mono-R/R	JNJ+AZA	JNJ+VEN	Mono-LR	Total
n	84	35	29	5	153
Patients who died during the study, *n* (%)	59[a] (70.2)	9 (25.7)	9 (31.0)	0 (0.0)	77 [a] (50.3)
Primary cause of death, *n* (%)					
AE	15 (17.9)	4 (11.4)	4 (13.8)	0 (0.0)	23 (15.0)
Related [b]	1 (1.2)	1 (2.9)	0 (0.0)	0 (0.0)	2 (1.3)
AE(s) unrelated	14 (16.7)	3 (8.6)	4 (13.8)	0 (0.0)	21 (13.7)
Progressive disease	43 (51.2)	4 (11.4)	4 (13.8)	0 (0.0)	51 (33.3)
Other	1 (1.2)	1 (2.9)	1 (3.4)	0 (0.0)	3 (2.0)
Patients who died within 30 days of last study treatment dose, *n* (%)	29 (34.5)	4 (11.4)	7 (24.1)	0 (0.0)	40 (26.1)
Primary cause of death, *n* (%)					
AE	15 (17.9)	3 (8.6)	4 (13.8)	0 (0.0)	22 (14.4)
Related [b]	1 (1.2)	1 (2.9)	0 (0.0)	0 (0.0)	2 (1.3)
AE(s) unrelated	14 (16.7)	2 (5.7)	4 (13.8)	0 (0.0)	20 (13.1)
Progressive disease	14 (16.7)	1 (2.9)	3 (10.3)	0 (0.0)	18 (11.8)
Other	0 (0.0)	0 (0.0)	0 (0.0)	0 (0.0)	0 (0.00)

Patients who died within 100 days of last study treatment dose, *n* (%)	47 (56.0)	9 (25.7)	9 (31.0)	0 (0.0)	65 (56.1)
Primary cause of death, *n* (%)					
AE	15 (17.9)	4 (11.4)	4 (13.8)	0 (0.0)	23 (15.0)
Related [b]	1 (1.2)	1 (2.9)	0 (0.0)	0 (0.0)	2 (1.3)
AE(s) unrelated	14 (16.7)	3 (8.6)	4 (13.8)	0 (0.0)	21 (13.7)
Progressive disease	31 (36.9)	4 (11.4)	4 (13.8)	0 (0.0)	39 (25.4)
Other	1 (1.2)	1 (2.9)	1 (3.4)	0 (0.0)	3 (2.0)

AE, adverse event; AML, acute myeloid leukaemia; AZA, azacitidine; CMML-2, chronic myelomonocytic leukaemia group 2; JNJ, JNJ-74856665; LR, lower risk; MDS, myelodysplastic syndrome; R/R, relapsed or refractory; TEAE, treatment-emergent AE; VEN, venetoclax.

[a] One patient had retroactive confirmation of death reported after data cut-off.

[b] An AE was categorized as related if assessed by the investigator as related to study agent.

No deaths were reported in the mono-LR arm.

Patients were counted only once for any given event, regardless of the number of times they actually experienced the event. The event experienced by the subject with the worst toxicity was used. If a patient had missing toxicity for a specific AE, the patient was only counted in the total column for that AE.

AEs were coded using Medical Dictionary for Regulatory Activities version 25.1.

Mono-R/R and JNJ+VEN: patients with R/R AML who had exhausted or were ineligible for standard therapeutic options, patients with newly transformed secondary AML who exhausted standard therapeutic options during treatment before transformation, and patients with high-risk or very high-risk, R/R MDS who exhausted or were ineligible for standard therapeutic options. The mono-R/R arm also was to include patients with R/R CMML-2 who exhausted or were ineligible for standard therapeutic options.

JNJ+AZA: patients with newly diagnosed or R/R AML who were unsuitable for intensive treatment with a curative intent (including stem cell transplantation) but eligible to receive AZA, patients with high-risk or very high-risk MDS; and patients with CMML-2.

Mono-LR: patients with very-low, low, or intermediate-risk MDS and transfusion dependence.

Preliminary clinical activity

Figure S7: Overall response and best overall response rates across treatment arms (all-treated/safety analysis population).

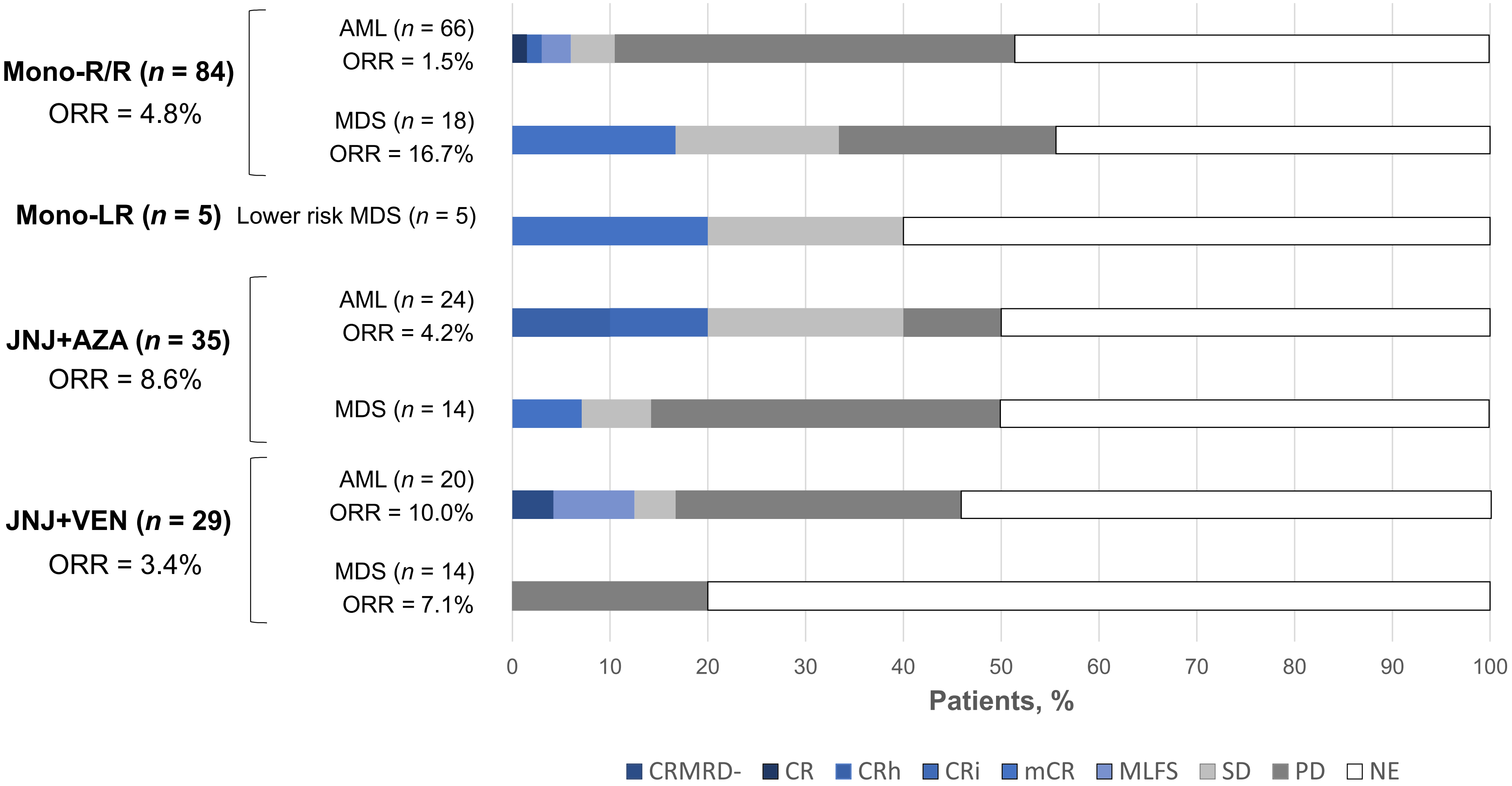

AML, acute myeloid leukaemia; AZA, azacitidine; CMML-2, chronic myelomonocytic leukaemia group 2; CR, complete response, CRh CR with partial haematologic recovery; CRi, CR with incomplete haematologic recovery; CRMDR, CR without minimal residual disease; JNJ, JNJ-74856665;

LR, lower risk; mCR marrow CR; MLFS, morphologic leukaemia-free state; MDS, myelodysplastic syndromes; NE, not evaluable; PD progressive disease; PR, partial response; RD, relapsed disease; R/R, relapsed or refractory; SD, stable disease; VEN, venetoclax.

CRh, CRi, and MLFS: responses for patients with an underlying diagnosis of AML; based upon modified European LeukemiaNet 2017 recommendations.[7,8] One patient in the mono-R/R arm met the criteria for CRh but was counted under CRi.

mCR: response for patients with an underlying diagnosis of MDS; based upon the International Working Group 2018 guidelines.[4,9] Two patients (1 in the mono-R/R and 1 the JNJ+AZA arm) met the criteria for mCR with haematologic improvement but were counted under mCR.

Response and overall response rate percentages were calculated using the total number of patients within the safety population within the relevant indication for a given cohort as the denominator. All other percentage calculations consider the total number of patients within the safety population for a given cohort as the denominator. The total number of patients with disease response collected may not sum to the total number of patients within the safety population.

Timing criteria were considered to determine best overall response. Response confirmation based on the duration of a response was required for CR and SD.

A responder was defined as a patient who achieved PR or better.

Best response at ≥1 post-baseline disease assessments (for patients who had ≥1 post-baseline disease assessment). Patients without a post-baseline disease assessment had their best response as “NE.”

Mono-R/R and JNJ+VEN: patients with R/R AML who had exhausted or were ineligible for standard therapeutic options, patients with newly transformed secondary AML who exhausted standard therapeutic options during treatment before transformation, and patients with high-risk or very high-risk, R/R MDS who exhausted or were ineligible for standard therapeutic options. The mono-R/R arm also was to include patients with R/R CMML-2 who exhausted or were ineligible for standard therapeutic options.

JNJ+AZA: patients with newly diagnosed or R/R AML who were unsuitable for intensive treatment with a curative intent (including stem cell transplantation) but eligible to receive AZA, patients with high-risk or very high-risk MDS; and patients with CMML-2.

Mono-LR: patients with very-low, low, or intermediate-risk MDS and transfusion dependence.

<u>Responder analyses</u>

In a responder analysis using best timepoint response, with no required duration, responses were seen in all treatment arms **(table below)**. Neither of the 2 treatment-naïve patients with AML (JNJ+AZA arm; **Table 1**) were responders; however, 1 (50.0%) had a bOR

of MLFS. Among 9 treatment-naïve patients with MDS (JNJ+AZA arm, 7; mono-LR arm, 2; **Table 1**), ORR was 11.1% (1/9). One JNJ+AZA-treated patient had a bOR of mCR; an additional patient (11.1% [1/9]; JNJ+AZA) had a bOR of SD.

Responder analysis using best timepoint responses.

Disease category	Patients dosed, *n* (%)	Response-evaluable population, *n* (%)	Responders, *n* (%)
**Mono-R/R (*n* = 84)**	84 (100.0)	73 (86.9)	7 (8.3)
MDS	18 (21.4)	15 (83.3)	4 (26.7)
AML	66 (78.6)	58 (87.9)	3 (5.2)
Primary AML (with myelodysplasia)	20 (23.8)	19 (95)	1 (5.3)
Primary AML (without myelodysplasia)	12 (14.3)	8 (66.7)	0 (0.0)
Secondary AML (history of MDS)	28 (33.3)	26 (92.9)	1 (3.8)
Secondary AML (other)	6 (7.1)	5 (83.3)	1 (20.0)
**JNJ+AZA (*n* = 35)**	35 (100.0)	30 (85.7)	4 (11.4)
CMML-2	1 (2.9)	0 (0.0)	0 (0.0)
MDS	14 (40.0)	11 (78.6)	2 (18.2)
AML	20 (57.1)	19 (95.0)	2 (10.5)
Primary AML (with myelodysplasia)	5 (14.3)	5 (100.0)	0 (0.0)
Primary AML (without myelodysplasia)	8 (22.9)	8 (100.0)	0 (0.0)
Secondary AML (history of MDS)	6 (17.1)	6 (100.0)	2 (33.3)
Secondary AML (other)	1 (2.9)	0 (0.0)	0 (0.0)
**JNJ+VEN (*n* = 29)**	29 (100.0)	26 (89.7)	2 (6.9)
MDS	5 (17.2)	3 (60.0)	0 (0.0)

AML	24 (82.8)	23 (95.8)	2 (8.7)
Primary AML (with myelodysplasia)	8 (27.6)	8 (100.0)	1 (12.5
Primary AML (without myelodysplasia)	5 (17.2)	5 (100.0)	1 (20.0)
Secondary AML (history of MDS)	8 (27.6)	8 (100.0)	0 (0.0)
Secondary AML (other)	3 (10.3)	2 (66.7)	0 (0.0)
**Mono-LR (*n* = 5)**	5 (100.0)	5 (100.0)	1 (20.0)
MDS	5 (100.0)	5 (100.0)	1 (20.0)

AE, adverse event; AML, acute myeloid leukaemia; AZA, azacitidine; CMML-2, chronic myelomonocytic leukaemia group 2; JNJ, JNJ-74856665; LR, lower risk; MDS, myelodysplastic syndrome; PR, partial response; R/R, relapsed or refractory; VEN, venetoclax.

A responder was defined as a patient who achieved PR or better for both AML and MDS. MLFS is excluded from the AML responder group. Best timepoint responses have no minimum duration of response, while best overall response requires that patients with MDS have evidence of a response for ≥4 weeks.

The response-evaluable population was defined as patients within the safety population with ≥1 post-baseline response evaluation by the investigator and ≥1 non-missing bone marrow biopsy/aspirate measurement at baseline.

Mono-R/R and JNJ+VEN: patients with R/R AML who had exhausted or were ineligible for standard therapeutic options, patients with newly transformed secondary AML who exhausted standard therapeutic options during treatment before transformation, and patients with high-risk or very high-risk, R/R MDS who exhausted or were ineligible for standard therapeutic options. Mono-R/R also was to include patients with R/R CMML-2 who exhausted or were ineligible for standard therapeutic options.

JNJ+AZA: patients with newly diagnosed or R/R AML who were unsuitable for intensive treatment with a curative intent (including stem cell transplantation) but eligible to receive AZA, patients with high-risk or very high-risk MDS; and patients with CMML-2.

Mono-LR: patients with very-low, low, or intermediate-risk MDS and transfusion dependence.

<u>Duration of response</u>

Median (SD) DOR among responders was 3.84 (1.15, not estimable) months (**figure and table below**).

Swimlane plot for duration of response among responders.

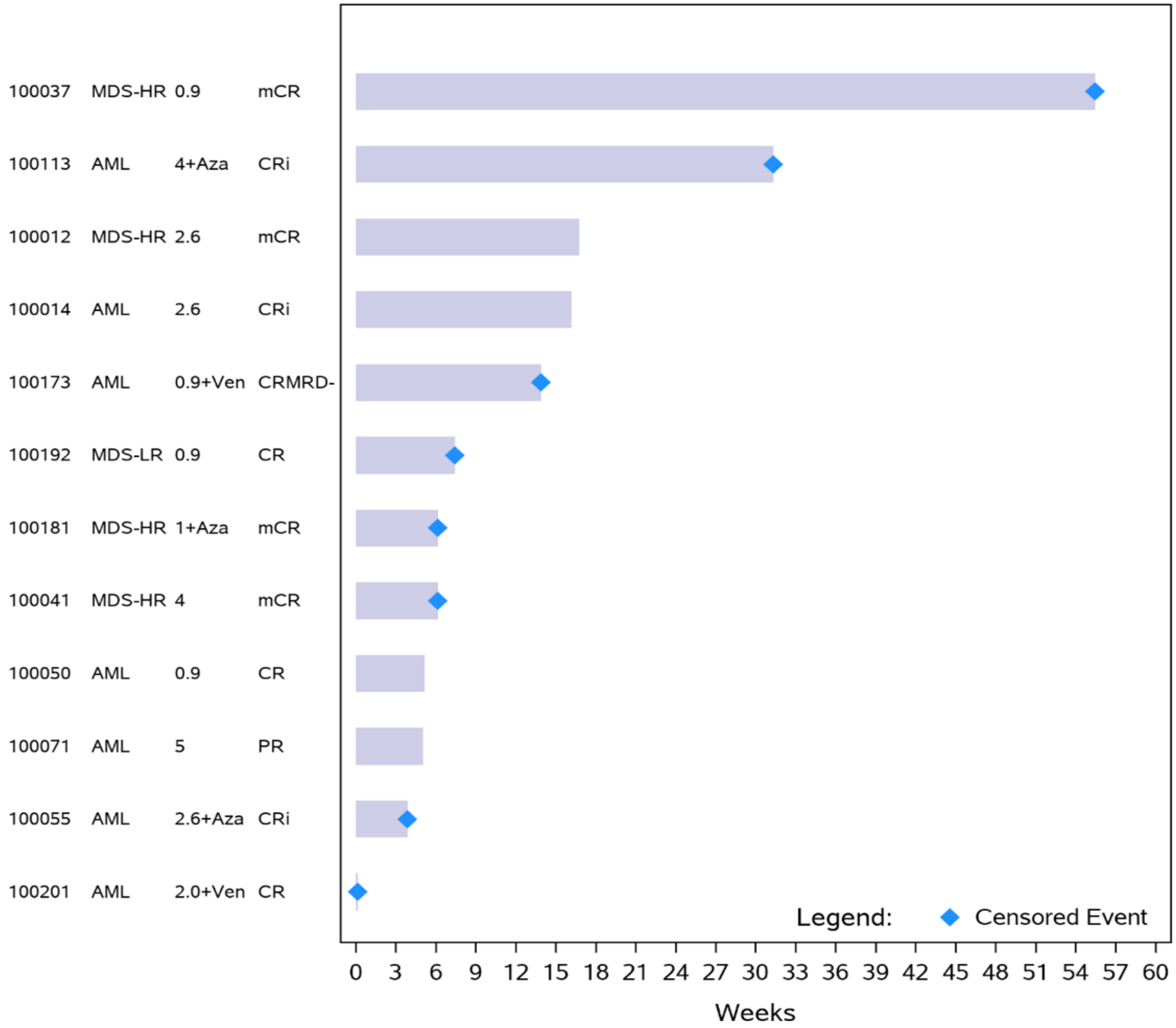

0.9, 0.9-mg JNJ-74856665; 1, 1.0-mg JNJ-74856665; 2.0, 2.0-mg JNJ-74856665; 2.6, 2.6-mg JNJ-74856665; 4, 4.0-mg JNJ-74856665; 5, 5.0-mg JNJ-74856665, AML, acute myeloid leukaemia; AZA, azacitidine; CR, complete response; CRMDR, CR without minimal residual disease; CRi, CR with incomplete haematologic recovery; *HR,* high risk; LR, lower risk; *mCR* marrow CR, MDS, myelodysplastic syndromes; PR, partial response;

VEN, venetoclax.

CRh, CRi, and MLFS: responses for patients with an underlying diagnosis of AML; based upon modified European LeukemiaNet 2017 recommendations.[7,8]

mCR: response for patients with an underlying diagnosis of MDS; based upon the International Working Group 2018 guidelines.[4,9]

A responder was defined as a patient who achieved PR or better.

Patient 100014 met the criteria for CRh, but was counted under CRi.

Patients 100012 and 100181 met the criteria for mCR with haematologic improvement, but was counted under mCR.

Duration of response based on investigator assessment

	**Mono-R/R**	**JNJ+AZA**	**JNJ+VEN**	**Mono-LR**	**Total**
n	6	3	2	1	12
DOR, months					
Events, *n* (%)	4 (66.7%)	0	0	0	4 (33.3%)
Censored events, *n* (%)	2 (33.3%)	3 (100.0%)	2 (100.0%)	1 (100.0%)	8 (66.7%)
Observed DOR range	(1.1, 12.7+)	(0.9+, 7.2+)	(0.0+, 3.2+)	(1.7+, 1.7+)	(0.0+, 12.7+)
Kaplan-Meier estimate, months					
25% quantile (95% CI)	1.18 (1.15, 3.84)	NE (NE, NE)	NE (NE, NE)	NE (NE, NE)	3.71 (1.15, 3.84)
Median (95% CI)	3.71 (1.15, NE)	NE (NE, NE)	NE (NE, NE)	NE (NE, NE)	3.84 (1.15, NE)
75% quantile (95% CI)	3.84 (1.18, NE)	NE (NE, NE)	NE (NE, NE)	NE (NE, NE)	NE (3.71, NE)

+, censored range value, AML, acute myeloid leukaemia; AZA, azacitidine; CI, confidence interval; CMML-2, chronic myelomonocytic leukaemia group 2; DOR, duration of response; JNJ, JNJ-74856665; LR, lower risk; MDS, myelodysplastic syndromes; NE, not estimable; R/R, relapsed or refractory; VEN, venetoclax.

A responder was defined as a patient who achieved PR or better for both AML and MDS. MLFS was excluded from the AML responder group. MDS responses must have persisted for ≥4 weeks to count as a response. For best timepoint response data, which has no minimum duration of response, refer to the **Responder analysis using best timepoint responses** table below.

Mono-R/R and JNJ+VEN: patients with R/R AML who had exhausted or were ineligible for standard therapeutic options, patients with newly transformed secondary AML who exhausted standard therapeutic options during treatment before transformation, and patients with high-risk or very high-risk, R/R MDS who exhausted or were ineligible for standard therapeutic options. Mono-R/R also was to include patients with R/R CMML-2 who exhausted or were ineligible for standard therapeutic options.
JNJ+VEN: patients with newly diagnosed or R/R AML who were unsuitable for intensive treatment with a curative intent (including stem cell transplantation) but eligible to receive AZA, patients with high-risk or very high-risk MDS; and patients with CMML-2.

Bone marrow blasts

Among patients with measurable disease burden at baseline and who were on treatment (AML, 54; MDS, 15), transient decreases in absolute BM blast values occurred in 26 (48.1%) with AML (range, -0.45 to -0.01) and 7 (46.7%) with MDS (-0.08 to -0.01) in the mono-R/R arm, 12 (22.2%) with AML (-0.79 to -0.005) and 7 (46.7%) with MDS (-0.13 to -0.006) in the JNJ+AZA arm, 16 (29.6%) with AML in the JNJ+VEN arm (-0.77 to -0.01), and 1 (6.7%) with MDS in the mono-LR arm (-0.01) (**figure and table below**).

Clinical activity, indicated by reduced BM blast involvement, was observed starting at 2.6-mg JNJ-74856665 in 2 patients with R/R AML (mono-R/R, 1; JNJ+AZA, 1). No DDIs were seen with AZA or VEN (or posaconazole), and toxicity did not increase with these combinations. The highest dose of JNJ-74856665 in both combination arms was 4.0 mg daily on weekdays only.

Best change in bone marrow blasts from baseline (all-treated population).

Mono-R/R

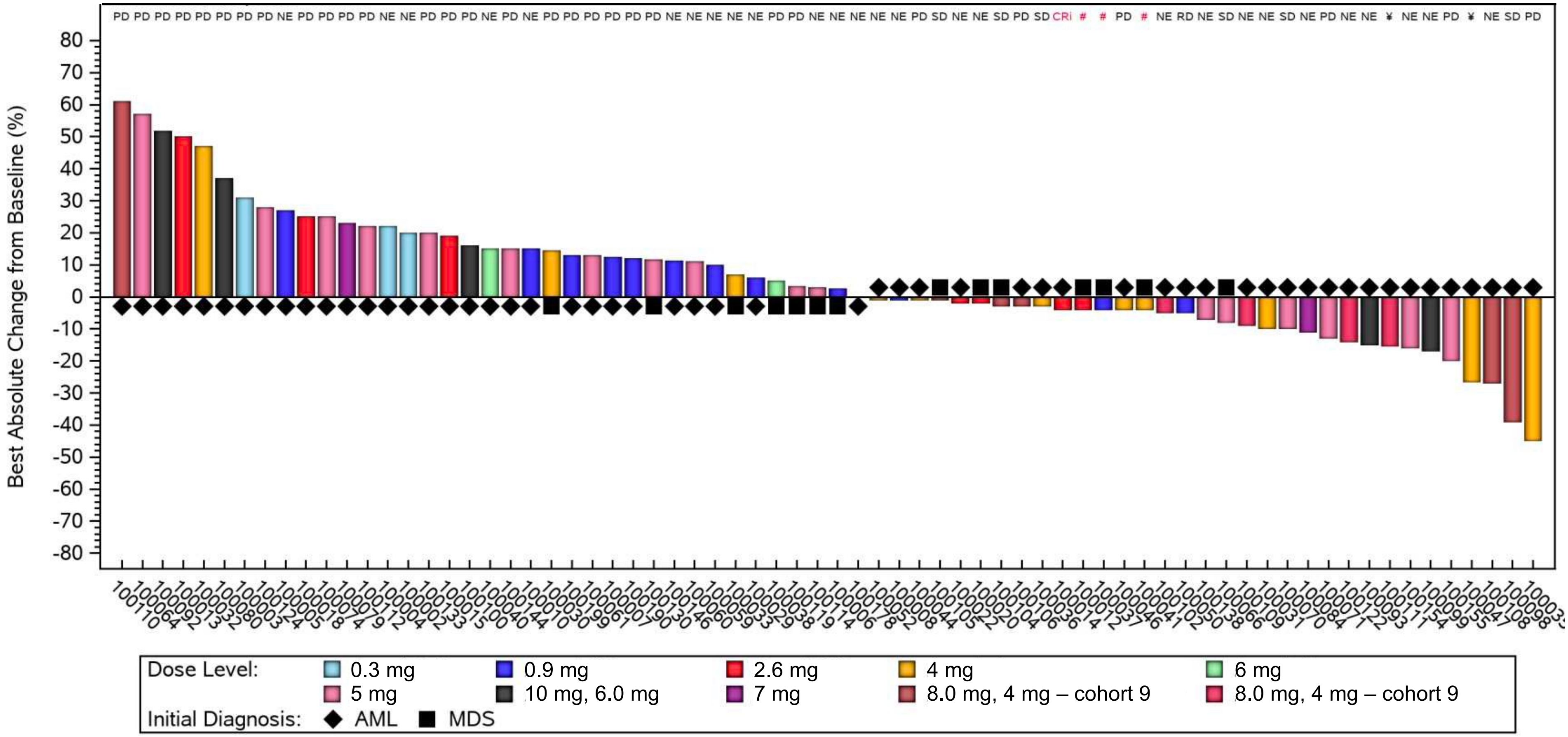

JNJ+AZA

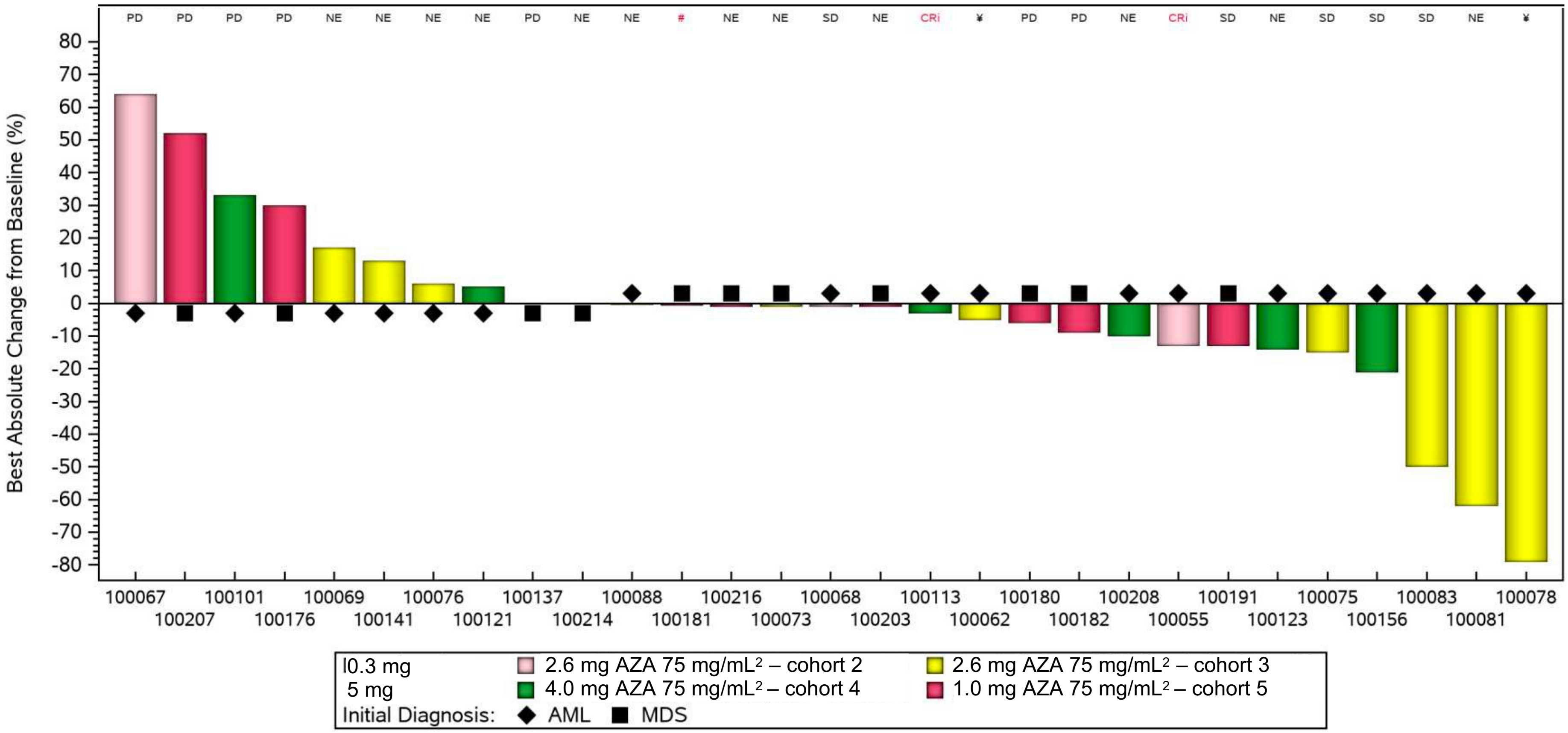
PD PD PD PD NE NE NE NE PD NE NE # NE NE SD NE CRi ¥ PD PD NE CRi SD NE SD SD SD NE ¥
Best Absolute Change from Baseline (%)
80 70 60 50 40 30 20 10 0 -10 -20 -30 -40 -50 -60 -70 -80
100067 100207 100101 100176 100069 100141 100076 100121 100137 100214 100088 100181 100216 100073 100068 100203 100113 100062 100180 100182 100208 100055 100191 100123 100075 100156 100083 100081 100078
l0.3 mg
5 mg
2.6 mg AZA 75 mg/mL2 – cohort 2
4.0 mg AZA 75 mg/mL2 – cohort 4
2.6 mg AZA 75 mg/mL2 – cohort 3
1.0 mg AZA 75 mg/mL2 – cohort 5
Initial Diagnosis: AML MDS

JNJ+VEN

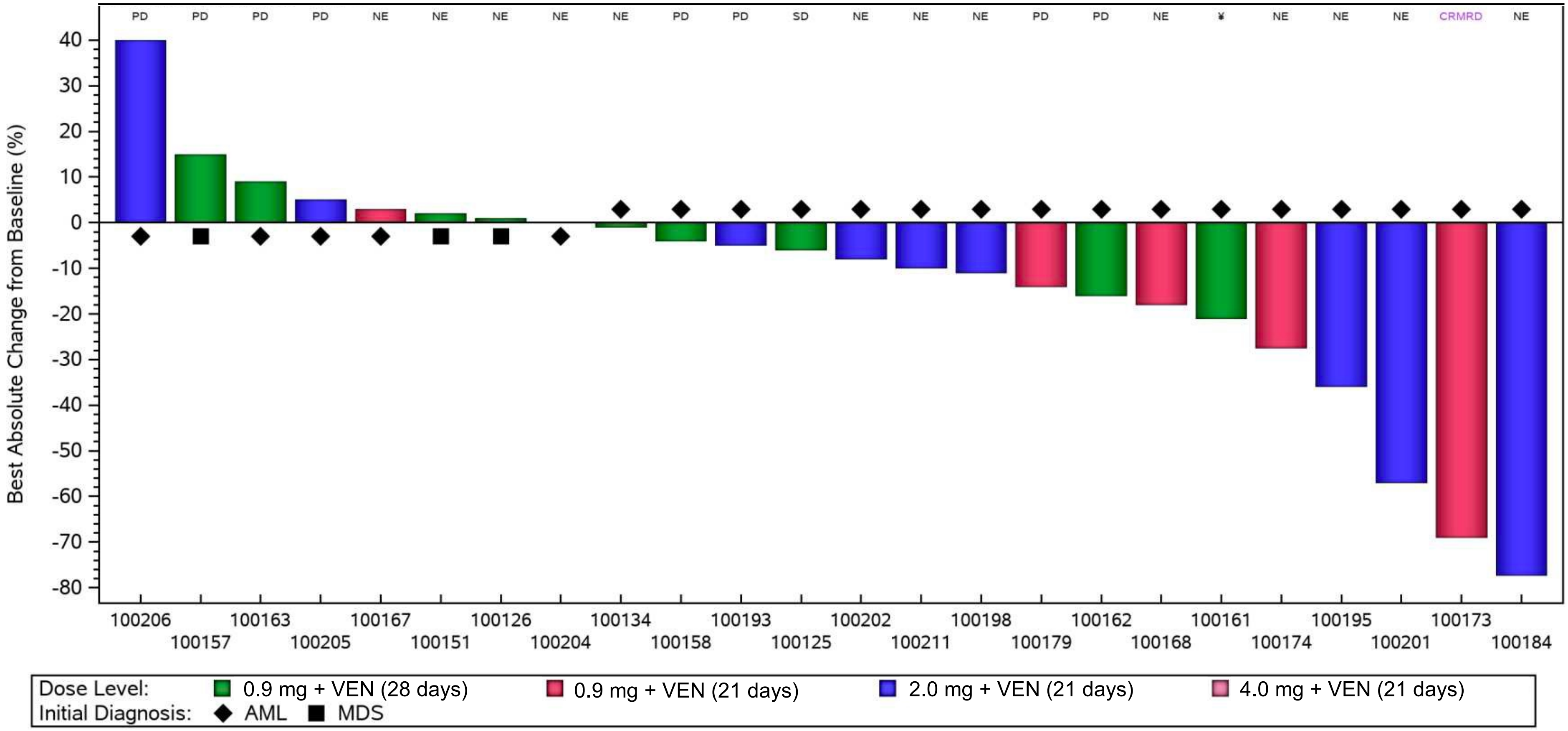

AML, acute myeloid leukaemia; AZA, azacitidine; CMML-2, chronic myelomonocytic leukaemia group 2; CR, complete response; CRh, CR with partial haematologic recovery; CRMRD, CR without minimal residual disease; CRi, CR with incomplete haematologic recovery; JNJ, JNJ-74856665; LR, lower risk; #, marrow CR (mCR); MLFS, morphologic leukaemia-free state; MDS, myelodysplastic syndromes; ¥, morphologic leukaemia-free state; NE not evaluable; *, pending response; PR, partial remission; R/R, relapsed or refractory; SD, stable disease; PD, progressive disease; RD, relapsed disease; VEN, venetoclax.

CRh, CRi, and MLFS: responses for patients with an underlying diagnosis of AML and is based upon modified European LeukaemiaNet 2017 recommendations.[7,8] One patient in the mono-R/R met the criteria for CRh but was counted under CRi.

mCR: response for patients with an underlying diagnosis of MDS and is based upon the International Working Group 2018 guidelines.[4,12] Two patients (1 in mono-R/R and 1 in JNJ+AZA) met the criteria for mCR with haematologic improvement but were counted under mCR.
Mono-R/R and JNJ+VEN: patients with R/R AML who had exhausted or were ineligible for standard therapeutic options, patients with newly transformed secondary AML who exhausted standard therapeutic options during treatment before transformation, and patients with high-risk or very high-risk, R/R MDS who exhausted or were ineligible for standard therapeutic options. Mono-R/R also was to include patients with R/R CMML-2 who exhausted or were ineligible for standard therapeutic options.
JNJ+VEN: patients with newly diagnosed or R/R AML who were unsuitable for intensive treatment with a curative intent (including stem cell transplantation) but eligible to receive AZA, patients with high-risk or very high-risk MDS; and patients with CMML-2.

Best transient decrease in bone marrow blasts (× 10^9) and change from baseline among patients with measurable disease burden at baseline (safety population).

	**Bone marrow aspirate/biopsy myeloblasts**						**Change from baseline**					
	n	Mean	SD	Med	Min	Max	*n*	Mean	SD	Med	Min	Max
AML												
Mon-R/R	26	0.28442	0.222892	0.25	0.02	0.73	26	-0.12462	0.11442	-0.1	-0.45	-0.01
JNJ+AZA	12	0.20292	0.192666	0.18	0	0.455	12	-0.22792	0.261425	-0.135	-0.79	-0.005
JNJ+VEN	16	0.18575	0.23823	0.076	0.01	0.92	16	-0.238	0.23881	-0.15	-0.773	-0.01
Mono-LR	0	-	-	-	-	-	0	-	-	-	-	-
MDS												
Mon-R/R	7	0.03786	0.029701	0.04	0.01	0.09	7	-0.03729	0.022171	-0.04	-0.08	-0.01
JNJ+AZA	7	0.05023	0.040236	0.05	0	0.1	7	-0.0451	0.049488	-0.01	-0.13	-0.0057
JNJ+VEN	0	-	-	-	-	-	0	-	-	-	-	-
Mono-LR	1	0	-	0	0	0	1	-0.01	-	-0.01	-0.01	-0.01

AML, acute myeloid leukaemia; AZA, azacitidine; JNJ, JNJ-74856665; LR, lower risk; MDS, myelodysplastic syndromes; med, median; min, minimum; max, maximum; n, number of patients with a non-missing value for the lab parameter at the specified diagnosis/arm; R/R, relapsed or refractory; SD, standard deviation; VEN, venetoclax.

Mono-R/R and JNJ+VEN: patients with R/R AML who had exhausted or were ineligible for standard therapeutic options, patients with newly transformed secondary AML who exhausted standard therapeutic options during treatment before transformation, and patients with high-risk or very high-risk, R/R MDS who exhausted or were ineligible for standard therapeutic options. Mono-R/R also was to include patients with R/R CMML-2 who exhausted or were ineligible for standard therapeutic options.

JNJ+VEN: patients with newly diagnosed or R/R AML who were unsuitable for intensive treatment with a curative intent (including stem cell transplantation) but eligible to receive AZA, patients with high-risk or very high-risk MDS; and patients with CMML-2.

Biomarker assessments

Figure S8: Maximum fold change in dihydroorotate from baseline by cohort and by occurrence of stomatitis (biomarker population).

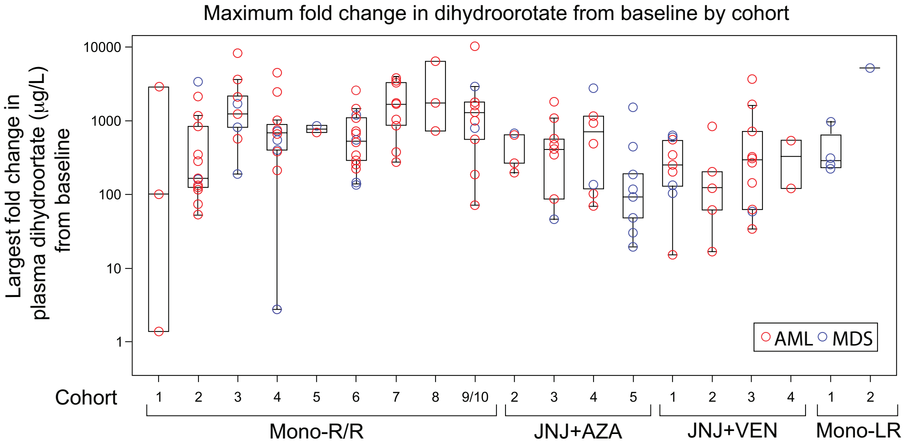

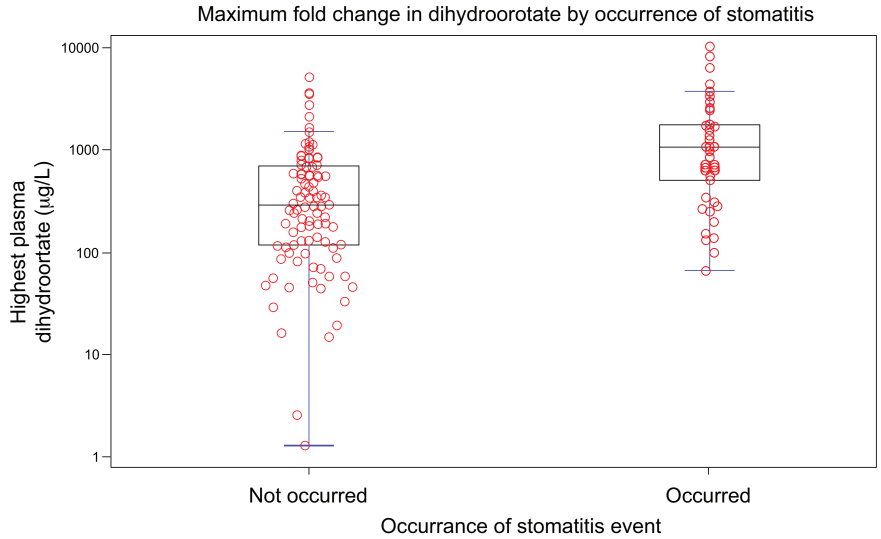

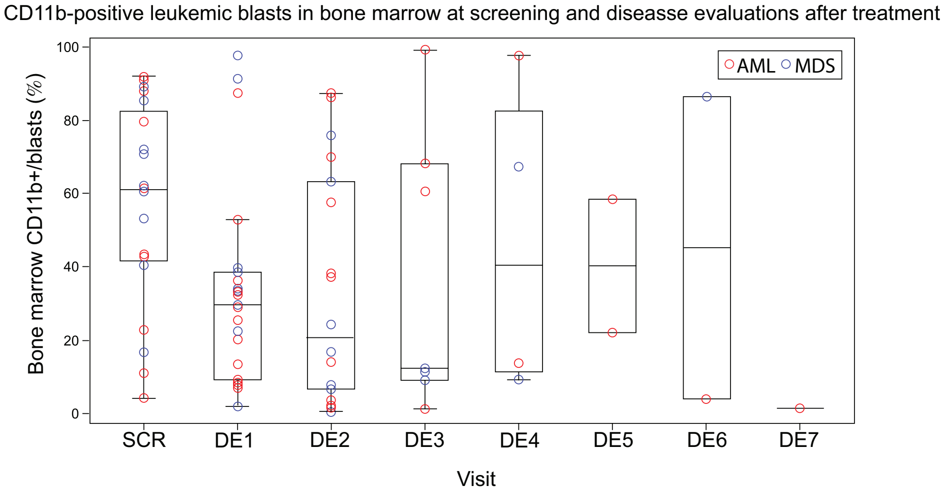

AML, acute myeloid leukaemia; AZA, azacitidine; CR, complete response; CRMDR, CR without minimal residual disease; CRi, CR with incomplete haematologic recovery; DE, disease evaluation; JNJ, JNJ-74856665; LR, lower risk; mCR marrow CR, MDS, myelodysplastic syndromes; PR, partial response; R/R, relapsed or refractory; SCR, screening; VEN, venetoclax.
CRh, CRi, and MLFS: responses for patients with an underlying diagnosis of AML; based upon modified European LeukaemiaNet 2017 recommendations.[7,8]
mCR: response for patients with an underlying diagnosis of MDS; based upon the International Working Group 2018 guidelines.[4,12]
Fold-change values on the y-axes of the dihydroorotate boxplots are displayed on the base 10 logarithmic scale.
Worst toxicity event experienced by the patient was used for evaluation of change in dihydroorotate from baseline by occurrence of stomatitis.
Mono-R/R: cohort 1, 0.3 mg; cohort 2, 0.9 mg; cohort 3, 2.6 mg; cohort 4, 4 mg; cohort 5, 6 mg; cohort 6, 5 mg; cohort 7, 10.6 mg; cohort 8, 7 mg; cohort 9 &10, 8.4 mg. JNJ+AZA: cohort 2, 2.6 mg + AZA 75 mg/m^2; cohort 3, 2.6 mg + AZA 75 mg/m^2; cohort 4, 4.0 mg + AZA 75 mg/m^2; cohort 5, 1.0 mg + AZA 75 mg/m^2; JNJ-74856665+VEN: cohort 1, 0.9 mg + VEN (28 days); cohort 2, 0.9 mg + VEN (21 days); cohort 3, 2.0 mg + VEN (21 days); cohort 4, 4.0 mg + VEN (21 days); mono-LR: cohort 1, 0.9 mg; cohort 2, 2.0 mg.
Mono-R/R and JNJ+VEN: patients with R/R AML who had exhausted or were ineligible for standard therapeutic options, patients with newly transformed secondary AML who exhausted standard therapeutic options during treatment before transformation, and patients with high-risk or very high-risk, R/R MDS who exhausted or were ineligible for standard therapeutic options. The mono-R/R arm also was to include patients with R/R CMML-2 who exhausted or were ineligible for standard therapeutic options.
JNJ+AZA: patients with newly diagnosed or R/R AML who were unsuitable for intensive treatment with a curative intent (including stem cell transplantation) but eligible to receive AZA, patients with high-risk or very high-risk MDS; and patients with CMML-2.
Mono-LR: patients with very-low, low, or intermediate-risk MDS and transfusion dependence.

Pharmacokinetic assessments

After oral, single and multiple JNJ-74856665 daily doses, maximum mean JNJ-74856665 plasma concentrations were reached within 2–8 hours, then concentrations decreased. Median t_{max} ranged from 2–5.53 hours (**table below**). Mean JNJ-74856665 plasma concentrations linearly increased with dose and appeared comparable across treatment arms. Mean C_{max}, AUC_{24h}, and AUC_{τ} generally increased with dose for all treatment arms, and mean dose-normalized values appeared comparable across cohorts and treatment arms, with overlapping ranges. Mean accumulation ratios ranged from 0.87–2.16 for C_{max} and 0.95–2.81 for AUC.

No DDIs were observed with overlapping ranges between JNJ-74856665 and AZA or VEN. After oral, single and multiple, daily doses of 0.9-mg JNJ-74856665 with or without posaconazole, plasma concentrations and PK parameters (mean C_{max}, AUC_{24h}, and AUC_{τ} after single dose) appeared comparable with overlapping ranges, though data were limited.

Pharmacokinetics of JNJ-74856665 after administration of JNJ-74856665 once daily per treatment arm (pharmacokinetic population).

	Cohort 1 0.3 mg	Cohort 2 0.3 mg	Cohort 3 0.3 mg	Cohort 4 0.3 mg	Cohort 5 0.3 mg	Cohort 6 0.3 mg	Cohort 7 0.3 mg	Cohort 8 0.3 mg	Cohort 9 0.3 mg	Cohort 10 0.3 mg
**Mono-R/R**										
C1D1, *n*	3	10	8 [a]	7 [c]	-	18	8	3	5	5
C_{max}, ng/mL	BQL (-)	5.70 (3.19)	17.2 (5.51)	30.3 (7.15)	-	39.3 (11.8)	66.5 (14.1)	44.5 (10.3)	63.3 (15.7)	67.0 (22.4)
t_{max}, h	-	5.09 (1.97-23.67)	3.10 (2.00-8.02)	3.92 (2.00-4.00)	-	2.21 (1.03-6.00)	4.16 (1.88-6.03)	2.00 (1.97-5.65)	3.93 (2.02-4.02)	2.08 (2.00-4.75)
AUC_{24h}, ng.h/mL	-	77.1 (44.9)	248 (62.8)	419 (115)	-	460 (124)	947 (313)	553 (5.58)	831 (321)	958 (629)
$C_{max, dn}$, ng/mL/mg	0.866 (-)	6.33 (3.54)	6.60 (2.12)	7.59 (1.79)	-	7.85 (2.35)	6.65 (1.41)	6.36 (1.47)	7.92 (1.96)	8.37 (2.81)
$AUC_{24h, dn}$, ng.h/mL/mg	2.54 (-)	85.7 (49.8)	95.3 (24.2)	105 (28.8)	-	92.0 (24.8)	94.7 (31.3)	79.1 0.797)	104 (40.2)	120 (78.6)
C2D1, *n*	-	6	3 [b]	4	-	12 [d]	-	-	-	-
C_{min}, ng/mL	-	4.93 (5.74)	11.0 (10.3)	8.87 (6.81)	-	7.49 (10.8)	-	-	-	-
C_{max}, ng/mL	-	10.8 (7.22)	37.9 (5.55)	39.6 (13.9)	-	44.2 (13.3)	-	-	-	-
t_{max}, h	-	3.03 (1.88-8.03)	4.00 (1.95-4.15)	2.02 (1.35-4.00)	-	2.14 (1.00-4.17)	-	-	-	-
AUC_t, ng.h/mL	-	175 (171)	-	463 (230)	-	575 (321)	-	-	-	-
AR_{Cmax}	-	1.96 (0.87)	194 (0.67)	1.36 (0.41)	-	1.19 (0.23)	-	-	-	-
AR_{AUC}	-	2.21 (0.77)	-	-	-	1.35 (0.47)	-	-	-	-
$C_{max, dn}$, ng/mL/mg	-	12.0 (8.02)	14.6 (2.14)	9.89 (3.48)	-	8.84 (2.65)	-	-	-	-

	Cohort 1 0.3 mg	Cohort 2 0.3 mg	Cohort 3 0.3 mg	Cohort 4 0.3 mg	Cohort 5 0.3 mg	Cohort 6 0.3 mg	Cohort 7 0.3 mg	Cohort 8 0.3 mg	Cohort 9 0.3 mg	Cohort 10 0.3 mg
$AUC_{24h, dn}$, ng.h/mL/mg	-	195 (189)	-	116 (57.6)	-	115 (64.2)	-	-	-	-
**JNJ+AZA**										
C1D1, *n*	-	5	5 [e]	4 [g]	5					
C_{max}, ng/mL	-	18.5 (4.42)	28.1 (11.4)	18.1 (4.68)	4.49 (1.70)					
t_{max}, h	-	2.20 (1.47-7.50)	2.03 (2.00-3.67)	4.14 (3.97-6.02)	5.53 (2.07-5.62)					
AUC_{24h}, ng.h/mL	-	220 (85.4)	292 (111)	243 (12.7)	61.5 (28.9)					
$C_{max, dn}$, ng/mL/mg	-	7.11 (1.70)	10.8 (4.40)	4.51 (1.17)	4.49 (1.70)					
$AUC_{24h, dn}$, ng.h/mL/mg	-	84.6 (32.8)	112 (42.5)	60.8 (3.18)	61.5 (28.9)					
C2D1, *n*	-	3	6 [f]	4	5 [h]					
C_{min}, ng/mL	-	BQL (-)	5.07 (7.25)	6.74 (7.10)	1.26 (1.68)					
C_{max}, ng/mL	-	13.9 (2.76)	24.4 (11.3)	33.4 (6.30)	7.64 (3.02)					
t_{max}, h	-	4.17 (4.13-5.00)	4.10 (2.07-6.00)	3.79 (1.00-5.67)	4.88 (0.92-5.53)					
AUC_t, ng.h/mL	-	154 (41.7)	372 (218)	472 (137)	112 (51.9)					
AR_{Cmax}	-	0.87 (0.20)	1.65 (0.99)	1.31 (0.40)	1.82 (0.86)					
AR_{AUC}	-	0.95 (0.11)	1.92 (0.73)	1.33 (0.37)	2.16 (1.08)					
$C_{max, dn}$, ng/mL/mg	-	5.33 (1.06)	9.37 (4.34)	8.36 (1.57)	7.64 (3.02)					
$AUC_{24h, dn}$, ng.h/mL/mg	-	59.1 (16.0)	143 (83.9)	118 (34.2)	112 (51.9)					
**JNJ+VEN**										
C1D1, *n*	10 [i]	6	11 [i]	-						
C_{max}, ng/mL	4.39 (1.93)	5.35 (2.32)	11.8 (5.08)	-						

	Cohort 1 0.3 mg	Cohort 2 0.3 mg	Cohort 3 0.3 mg	Cohort 4 0.3 mg	Cohort 5 0.3 mg	Cohort 6 0.3 mg	Cohort 7 0.3 mg	Cohort 8 0.3 mg	Cohort 9 0.3 mg	Cohort 10 0.3 mg
t_{max}, h	4.37 (1.92-5.88)	4.11 (2.00-24.00)	4.03 (2.03-24.00)	-						
AUC_{24h}, ng.h/mL	61.2 (28.6)	80.3 (42.2)	163 (93.0)	-						
$C_{max, dn}$, ng/mL/mg	4.87 (2.14)	5.91 (2.64)	5.88 (2.54)	-						
$AUC_{24h, dn}$, ng.h/mL/mg	68.0 (31.8)	88.7 (47.7)	81.3 (46.5)	-						
C2D1, *n*	8[j]	5	-	-						
C_{min}, ng/mL	2.26 (3.13)	3.27 (4.06)	-	-						
C_{max}, ng/mL	6.78 (2.53)	9.11 (4.85)	-	-						
t_{max}, h	4.00 (2.02-4.08)	4.00 (3.75-6.00)	-	-						
AUC_t, ng.h/mL	108 (69.1)	146 (67.5)	-	-						
AR_{Cmax}	1.98 (0.87)	2.16 (1.15)	-	-						
AR_{AUC}	1.97 (0.83)	2.81 (2.12)	-	-						
$C_{max, dn}$, ng/mL/mg	7.53 (2.81)	10.1 (5.39)	-	-						
$AUC_{24h, dn}$, ng.h/mL/mg	120 (76.8)	162 (75.0)	-	-						
**Mono-LR**										
C1D1, *n*	4	-								
C_{max}, ng/mL	5.33 (2.04) - tmax,	-								
t_{max}, h	3.07 (2.00-6.30)	-								
AUC_{24h}, ng.h/mL	65.5 (13.6)	-								

	Cohort 1<br>0.3 mg	Cohort 2<br>0.3 mg	Cohort 3<br>0.3 mg	Cohort 4<br>0.3 mg	Cohort 5<br>0.3 mg	Cohort 6<br>0.3 mg	Cohort 7<br>0.3 mg	Cohort 8<br>0.3 mg	Cohort 9<br>0.3 mg	Cohort 10<br>0.3 mg
$C_{max, dn}$, ng/mL/mg	5.92 (2.27)	-								
$AUC_{24h, dn}$, ng.h/mL/mg	72.8 (15.1)	-								
C2D1, *n*	4	-								
C_{min}, ng/mL	4.01 (3.23)	-								
C_{max}, ng/mL	10.6 (4.19)	-								
t_{max}, h	3.98 (2.20-4.08)	-								
AUC_t, ng.h/mL	161 (64.1)	-								
AR_{Cmax}	2.01 (0.52)	-								
AR_{AUC}	2.37 (0.56)	-								
$C_{max, dn}$, ng/mL/mg	11.8 (4.65)	-								
$AUC_{24h, dn}$, ng.h/mL/mg	178 (71.2)	-								

[a] *n* = 6 for AUC_{24h} and $AUC_{24h, dn}$.

[b] *n* = 4 for C_{min}.

[c] *n* = 5 for AUC_{24h} and $AUC_{24h, dn}$.

[d] *n* = 13 for C_{min}, *n* = 11 for AUC, AR_{AUC}, and $AUC_{\tau, dn}$.

[e] *n* = 4 for AUC_{24h} and $AUC_{24h, dn}$.

[f] *n* = 5 for AR_{AUC}.

[g] *n* = 3 for AUC_{24h} and $AUC_{24h, dn}$.

[h] *n* = 4 for AR_{AUC}.

[l] *n* = 8 for AUC_{24h} and $AUC_{24h, dn}$.

[j] *n* = 7 for AR_{AUC}.

AML, acute myeloid leukaemia; AR, accumulation ratio; AUC, area under to curve; AZA, azacitidine; BQL, below quantification limit (<0.500 ng/mL); $C_{max,}$ maximal drug concentration; $C_{min,}$ minimal drug concentration; CMML-2, chronic myelomonocytic leukaemia group 2; dn, dose normalized to 1 mg; JNJ, JNJ-74856665; LR, lower risk; MDS, myelodysplastic syndromes; R/R, relapsed or refractory; SD, standard deviation; t_{max}, time-to-peak drug concentration; VEN, venetoclax.

Mono-R/R and JNJ+VEN: patients with relapsed or refractory AML who had exhausted or were ineligible for standard therapeutic options, patients with newly transformed secondary AML who exhausted standard therapeutic options during treatment before transformation, and patients with high-risk or very high-risk, relapsed or refractory MDS who exhausted or were ineligible for standard therapeutic options. The mono-R/R arm also was to include patients with relapsed or refractory CMML-2 who exhausted or were ineligible for standard therapeutic options.

JNJ+AZA: patients with newly diagnosed or R/R disease AML who were unsuitable for intensive treatment with a curative intent (including stem cell transplantation) but eligible to receive AZA, patients with high-risk or very high-risk MDS; and patients with high CMML-2.

Mono-LR: patients with very low, low, or intermediate-risk MDS and transfusion dependence.

References

1. Van der Borght K, Tourny A, Bagdziunas R, et al. BIGL: Biochemically Intuitive Generalized Loewe null model for prediction of the expected combined effect compatible with partial agonism and antagonism. Sci Rep 2017;7(1):17935. DOI: 10.1038/s41598-017-18068-5.
2. Arber DA, Orazi A, Hasserjian R, et al. The 2016 revision to the World Health Organization classification of myeloid neoplasms and acute leukaemia. Blood 2016;127(20):2391-405. DOI: 10.1182/blood-2016-03-643544.
3. Greenberg PL, Tuechler H, Schanz J, et al. Revised international prognostic scoring system for myelodysplastic syndromes. Blood 2012;120(12):2454-65. DOI: 10.1182/blood-2012-03-420489.
4. Platzbecker U, Fenaux P, Ades L, et al. Proposals for revised IWG 2018 haematological response criteria in patients with MDS included in clinical trials. Blood 2019;133(10):1020-1030. DOI: 10.1182/blood-2018-06-857102.
5. Levey AS, Coresh J, Greene T, et al. Using standardized serum creatinine values in the modification of diet in renal disease study equation for estimating glomerular filtration rate. Ann Intern Med 2006;145(4):247-54. DOI: 10.7326/0003-4819-145-4-200608150-00004.

6. New York Heart Association. Criteria Committee., New York Heart Association. Nomenclature and criteria for diagnosis of diseases of the heart and great vessels. 8th ed. Boston: Little, Brown, 1979.

7. Dohner H, Estey E, Grimwade D, et al. Diagnosis and management of AML in adults: 2017 ELN recommendations from an international expert panel. Blood 2017;129(4):424-447. DOI: 10.1182/blood-2016-08-733196.

8. Bloomfield CD, Estey E, Pleyer L, et al. Time to repeal and replace response criteria for acute myeloid leukaemia? Blood Rev 2018;32(5):416-425. DOI: 10.1016/j.blre.2018.03.006.

9. Cheson BD, Greenberg PL, Bennett JM, et al. Clinical application and proposal for modification of the International Working Group (IWG) response criteria in myelodysplasia. Blood 2006;108(2):419-25. DOI: 10.1182/blood-2005-10-4149.

10. Savona MR, Malcovati L, Komrokji R, et al. An international consortium proposal of uniform response criteria for myelodysplastic/myeloproliferative neoplasms (MDS/MPN) in adults. Blood 2015;125(12):1857-65. DOI: 10.1182/blood-2014-10-607341.

11. Nagel D, Spranger S, Vincendeau M, et al. Pharmacologic inhibition of MALT1 protease by phenothiazines as a therapeutic approach for the treatment of aggressive ABC-DLBCL. Cancer Cell 2012;22(6):825-37. DOI: 10.1016/j.ccr.2012.11.002.

12. Cheson BD, Fisher RI, Barrington SF, et al. Recommendations for initial evaluation, staging, and response assessment of Hodgkin and non-Hodgkin lymphoma: the Lugano classification. J Clin Oncol 2014;32(27):3059-68. DOI: 10.1200/JCO.2013.54.8800.
